# NDR1-Dependent Regulation of Kindlin-3 Controls High-Affinity LFA-1 Binding and Immune Synapse Organization

**DOI:** 10.1128/MCB.00424-16

**Published:** 2017-03-31

**Authors:** Naoyuki Kondo, Yoshihiro Ueda, Toshiyuki Kita, Madoka Ozawa, Takashi Tomiyama, Kaneki Yasuda, Dae-Sik Lim, Tatsuo Kinashi

**Affiliations:** a Department of Molecular Genetics, Institute of Biomedical Science, Kansai Medical University, Hirakata, Osaka, Japan; b Division of Gastroenterology and Hepatology, Third Department of Internal Medicine, Kansai Medical University, Hirakata, Osaka, Japan; c Department of Urology and Andrology, Kansai Medical University, Hirakata, Osaka, Japan; d Department of Biological Sciences, National Creative Research Initiatives Center, Korea Advanced Institute of Science and Technology (KAIST), Daejeon, South Korea

**Keywords:** ICAM-1, immunological synapse, LFA-1, NDR1, Rap1, single-molecule measurement, vesicular transport

## Abstract

Antigen-specific adhesion between T cells and antigen-presenting cells (APC) during the formation of the immunological synapse (IS) is mediated by LFA-1 and ICAM-1. Here, LFA-1–ICAM-1 interactions were measured at the single-molecule level on supported lipid bilayers. High-affinity binding was detected at low frequencies in the inner peripheral supramolecular activation cluster (SMAC) zone that contained high levels of activated Rap1 and kindlin-3. Rap1 was essential for T cell attachment, whereas deficiencies of ste20-like kinases, Mst1/Mst2, diminished high-affinity binding and abrogated central SMAC (cSMAC) formation with mislocalized kindlin-3 and vesicle transport regulators involved in T cell receptor recycling/releasing machineries, resulting in impaired T cell-APC interactions. We found that NDR1 kinase, activated by the Rap1 signaling cascade through RAPL and Mst1/Mst2, associated with and recruited kindlin-3 to the IS, which was required for high-affinity LFA-1/ICAM-1 binding and cSMAC formation. Our findings reveal crucial roles for Rap1 signaling via NDR1 for recruitment of kindlin-3 and IS organization.

## INTRODUCTION

T cells are highly motile within lymphoid tissues, as they scan rare antigens on dendritic cells. When T cells encounter cognate antigens, they decelerate and exhibit distinct adhesive behaviors characterized by initial brief migratory interactions that are followed by long-lasting interactions. Once T cells are fully activated, they undergo several rounds of proliferation and become highly motile (1). The phases of T cell and antigen-presenting cell (T-APC) dynamics are modulated by a number of parameters, including antigen dose and strength of T cell receptor (TCR) stimulation, which also influence the outcome of T cell activation and the acquisition of effector/memory functions (2). An *in vivo* imaging study revealed a requirement for LFA-1 and ICAM-1 for T cell arrest on APCs and memory responses (3), suggesting that TCR signals control LFA-1 adhesiveness. However, the mechanisms that regulate LFA-1 and ICAM-1 binding in phased T-APC interactions remain unclear.

Antigen-specific T-APC interactions have been extensively studied in the immunological synapse (IS), which is composed of a central supramolecular activation cluster (cSMAC) of TCR-peptide major histocompatibility complex (pMHC) surrounded by a peripheral ring of LFA-1/ICAM-1 and associated talin (pSMAC) and a distal supramolecular activation cluster (dSMAC) of F-actin (4–6). The dynamic formation of the cSMAC and pSMAC was revealed using a supported planar lipid bilayer (SPLB) incorporating pMHC complexes and ICAM-1 (5). Total internal reflection fluorescence (TIRF) microscopy demonstrated that the agonist pMHC induced a constant generation of peripheral TCR microclusters with sustained active TCR signaling that were transported into the center of the structure (7, 8). The cSMAC has been increasingly recognized as the site of signal termination (7, 8), endocytosis of engaged TCR, and targeted secretion (8, 9). TCR/CD3 complexes were recycled to the IS using intraflagellar and vesicle transport components (10, 11) and released to the extracellular space of the cSMAC as TCR-enriched microvesicles in an ESCRT (endosomal sorting complex required for transport)-dependent manner (12).

Compared to TCR-pMHC interactions, our understanding of the regulatory mechanisms for LFA-1/ICAM-1 binding within the IS is still limited. TCR ligation triggers rapid activation of LFA-1 via inside-out signaling (13) and shifts the equilibrium of LFA-1 conformations from low/intermediate to high affinity for ICAM-1, and it initiates cell surface clustering (14, 15). Inside-out signaling activates the key integrin activators talin and kindlin-3 (16–19), which interact with integrin cytoplasmic regions, leading to enhanced LFA-1 ligand-binding affinity (16). Ligand binding induces/stabilizes high-affinity conformations of LFA-1 as well as triggers outside-in signaling to activate integrin-dependent functions (20). TCR-stimulated T cells that were deficient for talin1 failed to adhere through LFA-1/ICAM-1 (21). In T cells, kindlin-3 is required for stabilization of LFA-1/ICAM-1 following TCR triggering (22) and during extravasation (23). It is generally thought that inside-out signals cause direct binding of talin-1 and kindlin-3 to the tail region of the β subunits, leading to a separation of α/β integrin cytoplasmic tails, which induces conformational changes to the stalk and headpiece regions, resulting in a shift from bent low-affinity to extended intermediate- and high-affinity conformations (16, 20, 24, 25). It is still unclear how heterogeneous binding events of LFA-1 and ICAM-1 are regulated by inside-out signals and IS formation through talin and kindlin-3.

The small GTPase Rap1 is a potent activator of integrins, including LFA-1 (26). We previously demonstrated that mammalian Hippo kinase Mst1 was associated with and activated by the Rap1-GTP binding protein RAPL, which in turn formed a complex with and activated LFA-1 (27, 28). Furthermore, ADAP/SKAP1 formed a complex with Mst1 and RAPL (29) or with RIAM and talin (30). Interestingly, lymphocytes and thymocytes from Mst1-deficient mice had impaired LFA-1-dependent adhesion and migration and exhibited defective self-tolerance (28, 31–33). Mst1/Mst2-deficient mice also exhibited aggravated trafficking phenotypes (34). The emerging roles of Mst1/Mst2 in lymphocyte trafficking, adhesion, and cell polarity are distinct from the canonical Hippo-LATS-YAP pathway to restrain cell proliferation and are consistent with phenotypes of *MST1* mutations identified in human immunodeficiencies with recurrent infection and autoantibody production (35). Recently, several regulators downstream of Mst1/Mst2 that mediate lymphocyte trafficking were reported, including DOCK8 (34), Rab13 (36), and LATS homolog NDR kinases (37). However, the role of TCR-triggered Rap1 signaling to Mst1 for LFA-1 activation and formation of the IS remains unknown. To date, there is limited information regarding the dynamics and regulation of LFA-1/ICAM-1 binding events for intracellular signaling during IS development in primary T cells recognizing physiological ligands.

We analyzed LFA-1/ICAM-1 binding dynamics on supported lipid bilayers presenting pMHC and clarified the role of Rap1 signaling during IS formation at the single-molecule level. High-affinity binding preferentially occurred at low frequencies in the inner pSMAC zone enriched for active Rap1 and kindlin-3. Deficiencies of Rap1 and Mst1/2 diminished high-affinity binding and abrogated cSMAC formation, which was characterized by mislocalization of kindlin-3 and vesicle transport regulators. Depletion of NDR1 severely impaired IS formation and kindlin-3 accumulation with reduced high-affinity binding. Our findings reveal crucial roles for Rap1 signaling via NDR1 to recruit kindlin-3 and IS formation.

## RESULTS

### Single-molecule imaging of ICAM-1 binding events in immunological synapses.

**FIG 1:**
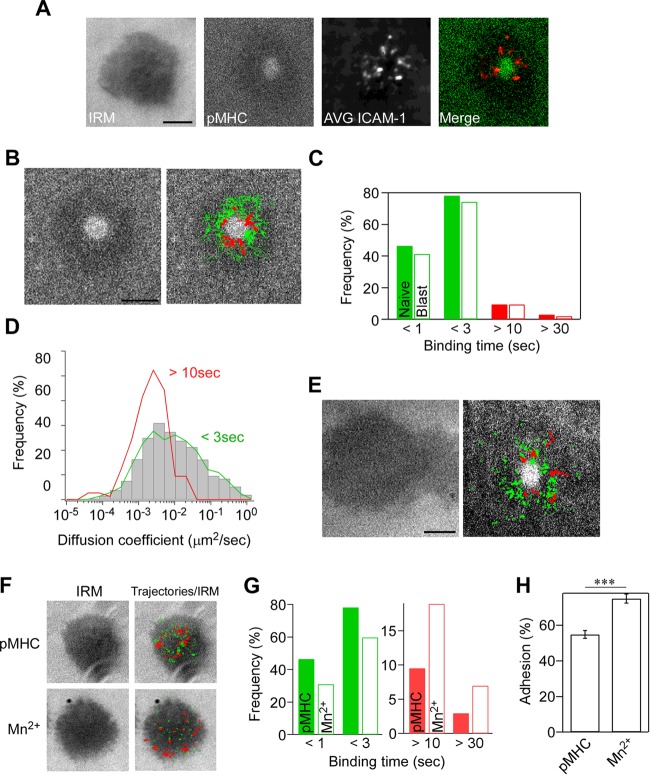
Single-molecule measurement of ICAM-1 binding to LFA-1. Naive OT-II T cells were incubated on supported planar lipid bilayers presenting ATTO-647N-labeled ICAM-1-GPI (300 molecules/μm^2^) and ovalbumin peptide (OVA_323–339_) complexed with I-A^b^ (200 molecules/μm^2^). Images were recorded at 30 frames/s using a TIRF microscope. (A) Representative images of interference reflection microscopy (IRM) and pMHC, averaged time-lapse images of ICAM-1 (6,000 frames) (AVG ICAM-1), and a merged image of pMHC (green) with averaged ICAM-1 (red). Scale bar, 2.5 μm. (B) Trajectories of individual bound ICAM-1 spots in IS of naive OT-II T cells on lipid bilayers presenting pMHC. Trajectories with a lifetime of more than 0.5 s (green) and 10 s (red) are shown. Scale bar, 2.5 μm. (C) Lifetime profiles of ICAM-1 binding obtained from naive OT-II T cells (closed bar) and OT-II T cell blasts (open bar). The calculated two-way chi-square *P* value was 0.723. (D) Profiles of diffusion coefficients (*D*) of trajectories for bound ICAM-1 with lifetimes of less than 3 s (low-intermediate) and longer than 10 s (high). Gray bars indicate *D* of all trajectories. Data from 842 individual trajectories of at least 15 synapses are shown. The difference between *D* of high and low-intermediate is statistically significant (*P* < 0.0001 by Mann-Whitney test). (E) Trajectories of individual ICAM-1 spots in IS of OT-II T cell blasts as described for panel B. (F to H) The effect of Mn^2+^ versus pMHC on single-molecule LFA-1–ICAM-1 interactions. (F) Images of IRM alone and merged IRM plus trajectories of LFA-1-bound ICAM-1 with lifetimes of more than 0.5 s (green) and 10 s (red). (G) Lifetime of ICAM-1 binding with (closed bar) or without (open bar) 1 mM Mn^2+^. (H) Comparison of the attachment frequency with or without Mn^2+^. Percentages of attached cells were calculated based on the IRM images for multiple fields (***, *P* < 0.0001 by Mann-Whitney test).

Previous studies of LFA-1 in IS relied on conformation/activation epitopes of LFA-1 or lateral mobility of ICAM-1 (20, 38). Although previous findings suggested that LFA-1 conformational changes and clustering events were organized during IS formation, *in situ* measurements of the binding kinetics between LFA-1 and ICAM-1 have not yet been reported. Using antibodies (Ab) to detect the conformation/activation epitopes of LFA-1 limits the types of studies that can be performed. To overcome the limitations of using antibodies, we directly measured the binding kinetics of LFA-1–ICAM-1 interactions at the single-molecule level by taking advantage of lateral mobility decelerations of surface receptors upon ligand binding on supported lipid bilayers, which was successfully used to assess TCR-pMHC interactions (39). ICAM-1-GPI was labeled with a diluted photoresistant dye prior to incorporation into supported lipid bilayers and imaged in real time using TIRF microscopy (see Fig. S1A to D and Video S1 in the supplemental material). When ovalbumin (OVA)-specific naive OT-II T cells were incubated on OVA-peptide MHC (pMHC)-loaded bilayers, freely mobile ICAM-1 was frequently arrested in cell-attached areas (Video S2). Averaged images of 6,000 frames (200 s) revealed slow or stably arrested ICAM-1 spots that were localized exclusively outside the cSMAC, with high-intensity spots often found near the cSMAC (Fig. 1A). Diffusion coefficients of arrested ICAM-1 were two orders smaller than those of ICAM-1 in cell-free areas (Fig. S1E and G), suggesting that slow-mobility spots represented ICAM-1 captured by LFA-1. This was largely supported by single-particle tracking (SPT) analyses using LFA-1-deficient OT-II T cells (Fig. S1F). The lower limit of detection for arrest events by SPT was 0.5 s with a 99% cutoff value. The upper limit of detection for binding events was set at 200 s based on photostability under our imaging laser power (half-life [*t*_1/2_] of 3 min).

Trajectories of engaged ICAM-1 with durations of longer than 0.5 s from multiple IS were overlaid with cSMAC images for over 1,000 trajectories (Fig. 1B and Video S2). ICAM-1 binding occurred outside the cSMAC with a binding duration of up to 175 s. Approximately half of the engaged ICAM-1 had a binding duration of less than 1 s, while 20 to 30% ranged from 1 to 3 s (Fig. 1C). The semilogarithmic plot of ICAM-1 binding event numbers (*n* = 912) against lifetimes suggests a sum of multiple exponential curves (Fig. S1H). When fitted for long binding durations with single exponential curves, the dissociation rate constant (*k*_off_) was 0.032 s^−1^ after photobleaching correction (average lifetime, 31.2 s), with a high correlation for lifetimes longer than 10 s (correlation coefficient [*r*^2^] of 0.99). The calculated off-rate was in good agreement with that of high-affinity conformers according to the study using recombinant locked high-affinity conformers of the αL I domain (*k_off_* of 0.014 to 0.045 s^−1^, lifetime of 22 to 71 s) (40) or intact LFA-1 (*k_off_* of 0.0298 s^−1^, lifetime of 33.5 s) (41). Since the off-rate is a primary determinant to distinguish high-affinity conformers from intermediate-affinity conformers (0.43 to 0.77 s^−1^, lifetime of 1.3 to 2.3 s) (40), ICAM-1 binding events with lifetimes longer than 10 s likely are mediated by high-affinity interactions. Since the major difference between intermediate- and low-affinity interactions is the on-rate (40), our method could not distinguish the interactions of intermediate affinity from low affinity based on the off-rate. Thus, the binding duration for most engaged ICAM-1 would be mediated by low/intermediate-affinity interactions between LFA-1 and ICAM-1, and these interactions were broadly distributed (Fig. 1B, green). ICAM-1 binding events with lifetimes longer than 10 s were defined here as high-affinity interactions and were detected at a frequency of less than 10% (Fig. 1C), and they were located near the cSMAC (Fig. 1B, red). ICAM-1 spots that engaged longer than 10 s were almost sessile (*D* = 1.9 × 10^−3^ μm^2^/s), while engaged ICAM-1 spots with a lifetime of less than 3 s were more mobile (*D* = 2.6 × 10^−2^ μm^2^/s) (Fig. 1D). Previous studies showed that LFA-1 lateral mobility on the plasma membrane exhibited a large heterogeneity of diffusion coefficients, ranging from 10^−3^ to 10^−1^ μm^2^/s. ICAM-1 binding increased a fraction with low mobility (42–44), which was comparable to those of bound ICAM-1 of longer than 10 s in IS.

Since we failed to detect centripetal movements of engaged ICAM-1, in contrast to prior reports of Jurkat T cells (45), we also examined cultured OT-II T cell blasts under the same conditions. We found that OT-II T cell blasts spread more extensively than naive T cells, and some trajectories exhibited centripetal movements (Fig. 1E and Video S3), suggesting that centripetal migration of engaged ICAM-1 occurs in F-actin-rich, activated T cells. Nonetheless, lifetime profiles of engaged ICAM-1 on T cell blasts were similar to those with naive T cells (*P* = 0.723) (Fig. 1C). We also examined the induction of high-affinity conformations using manganese ions (Mn^2+^), which are strong activators of integrins. The addition of Mn^2+^ stimulated T cell attachment on bilayers at a similar ICAM-1 density in the absence of pMHC (Fig. 1F). As expected, Mn^2+^ addition resulted in a 2-fold increase in ICAM-1 binding durations longer than 10 s with a reciprocal decrease of ICAM-1 binding durations shorter than 3 s (*P* < 0.0001) (Fig. 1G), as well as increased attachment (*P* < 0.0001) (Fig. 1H). Stably engaged ICAM-1 molecules in the presence of Mn^2+^ were immobile and broadly distributed, in contrast to IS containing pMHC, suggesting that intracellular signaling triggered by an engaged TCR organizes LFA-1 and ICAM-1 binding.

### Requirement of Rap1 and Mst1/Mst2 in both pSMAC and cSMAC development.

**FIG 2:**
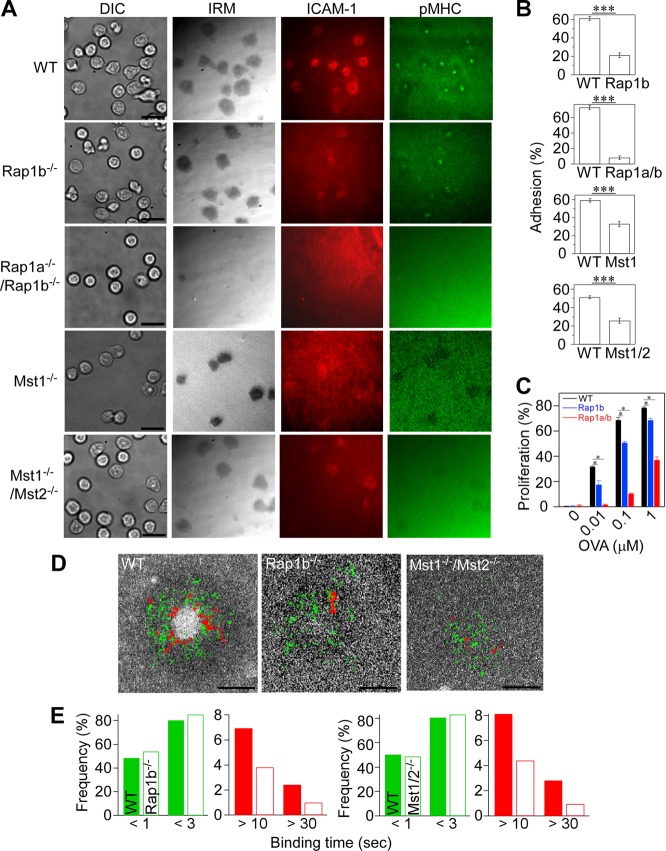
Impaired IS formation in naive OT-II T cells deficient for Rap1 and Mst1/Mst2. (A) IS formation of naive OT-II T cells isolated from mutant mice deficient for Rap1b, Rap1a/Rap1b, Mst1, and Mst1/Mst2. Scale bar, 10 μm. TIRF microscopy was used to image IS formation on lipid bilayers displaying pMHC and ICAM-1. (B) Attachment of naive wild-type T cells (WT) and naive T cells from *Rap1b*^−/−^ (Rap1b), *Rap1a*^−/−^/*Rap1b*^−/−^ (*Rap1a/b*), *Mst1*^−/−^, and *Mst1*^−/−^/*Mst2*^−/−^ (Mst1/2) mice. Percentages of attached cells with IRM images larger than 10 μm^2^ from multiple fields were counted. ***, *P* < 0.0001. (C) Antigen-dependent proliferation of wild-type and mutant OT-II T cells. Fractions of proliferating cells were determined by CFSE dilution after coculture with bone marrow-derived dendritic cells in the presence of OVA_323–339_ peptides as indicated. *, *P* < 0.01. (D) Single-molecule trajectories of ICAM-1 overlaid on cSMAC. Trajectories with lifetimes longer than 0.5 s (green) and 10 s (red) are shown. Scale bar, 2.5 μm. (E) Profiles of ICAM-1 binding in wild-type and *Rap1b*^−/−^ or *Mst1*^−/−^/*Mst2*^−/−^ OT-II cells. Short binding and long binding events are colored in green and red, respectively. The two-way chi-square *P* value using the *Rap1b*^−/−^ or *Mst1*^−/−^/*Mst2*^−/−^ data sets was <0.0001 or 0.0132, respectively.

To explore the roles of Rap1 signaling in IS formation, we next examined whether Rap1 and Mst1/Mst2 were required for IS formation using antigen-specific naive T cells from mutant OT-II mice. We used *Rap1b*^*f/f*^ and *Rap1a*^−/−^; *Rap1b*^*f/f*^ mice that were crossed with OT-II mice expressing cre recombinase driven by a CD4 promoter to inactivate the Rap1 gene in T cells (46). In *Rap1b*^*f/f*^; *CD4-cre*; *OT-II* mice, total Rap1 protein levels were reduced to less than 10% of wild-type OT-II T cell levels (46). Compared to naive wild-type OT-II T cells, naive *Rap1b*^*f/f*^; *CD4-cre*; *OT-II* T cells (*Rap1b*^−/−^; Rap1b knockout [KO]) exhibited immature IS with poor ICAM-1 ring formation and small central pMHC clusters (Fig. 2A), as well as reduced adhesion on lipid bilayers (Fig. 2B). The targeting of both *Rap1a* and *Rap1b* in T cells (*Rap1b*^*f/f*^; *CD4-cre*; *Rap1a*^−/−^; *OT-II*) (Rap1 double knockout [DKO]) almost abolished IS formation and cell attachment (Fig. 2A and B). Naive *Mst1*^−/−^ *OT-II* T cells failed to form SMAC and had reduced adhesion levels (Fig. 2A and B). OT-II T cells deficient for both Mst1 and Mst2 (Mst1/2 DKO) had further reduced adhesion with an impairment of IS formation (Fig. 2A and B). In line with defective IS formations on lipid bilayers, antigen-dependent proliferation was also impaired in Rap1b KO, Rap1 DKO (Fig. 2C), Mst1 KO, and Mst1/2 DKO OT-II T cells (Fig. S2A). Understanding that the Rap1-GTP-binding protein RAPL activates Mst1 in T cells (27), we sought to determine the effect of RAPL knockdown in OT-II T cells. Lentiviral transduction of short hairpin RNA (shRNA) specific for RAPL reduced its protein levels by more than 70% in cultured T cells. RAPL knockdown caused defective mature IS formation (Fig. S2B and C). T cell attachment was less impacted by Mst1/2 DKO or RAPL depletion than Rap1 DKO T cells, suggesting that RAPL and Mst1/2 are required in part in Rap1-dependent adhesion to ICAM-1.

To further elucidate the regulatory mechanism in detail, we measured LFA-1/ICAM-1 binding kinetics of naive OT-II T cells derived from mutant mice. *Rap1b*^−/−^ T cells had short ICAM-1 binding events that were comparable to those of wild-type T cells, but stable binding events longer than 10 s were significantly decreased (*P* < 0.0001) (Fig. 2D and E). Similarly, Mst1/2 DKO T cells exhibited decreased stable bindings longer than 10 s (*P* = 0.0132) (Fig. 2D and E). We were unable to determine ICAM-1 binding profiles in Rap1 DKO due to a paucity of binding events, indicating that short binding events were also severely affected. Although Rap1 and Mst1/Mst2 are required for IS formation, Mst1/Mst2 exerted the greatest effect on high-affinity binding events.

We next investigated the impact of impaired IS formation with reduced high-affinity binding of Mst1 and Mst2 deficiencies on T cell-dendritic cell (T-DC) interactions in lymphoid tissues. Two-photon microscopy was used to capture live images of naive OT-II T cells that were applied to the surface of cut peripheral lymph nodes (LN) in which OVA-pulsed DC had been adoptively transferred. In the 30-min imaging time frame, the median T-DC contact duration for wild-type T cells was 4.1 min. Application of an inhibitory anti-LFA-1 antibody decreased contacts with duration lasting longer than 10 min and increased transient contacts with a median duration of 2.8 min (Fig. S2D). Simultaneous imaging of wild-type and Mst1/2 DKO T cells revealed a severe reduction of stable T-DC contacts lasting longer than 10 min in Mst1/2 DKO T cells with a median contact time of 1.4 min (Fig. S2E). Taken together with the single-molecule data, these results indicate that high-affinity binding events of LFA-1 and ICAM-1 regulated by Mst1/Mst2 require stable contact of antigen-dependent interactions with DC.

### Distribution of activated Rap1 and integrin regulators in IS.

To determine whether high-affinity interactions of LFA-1 and ICAM-1 that localized at the inner area of the pSMAC are regulated via Rap1 signaling, we examined the distribution of activated Rap1 in naive OT-II T cells using a green fluorescent protein (GFP)-tagged Rap affinity probe (RalGDS-RBD) specific for GTP-bound Rap1 and Rap2 (47). First, the specificity of the probe was validated using T cells derived from *Rap1b*^−/−^ mice. The Rap affinity probe was diffusely distributed in the cytoplasm of both wild-type and *Rap1b*^−/−^ T cells without stimulation (Fig. S3A). Importantly, when stimulated with anti-CD3 antibodies, the probe was relocated to the plasma membrane of wild-type T cells but not Rap1b-deficient T cells (Fig. S3A). Therefore, the Rap affinity probe was a reporter of Rap1 activation.

**FIG 3:**
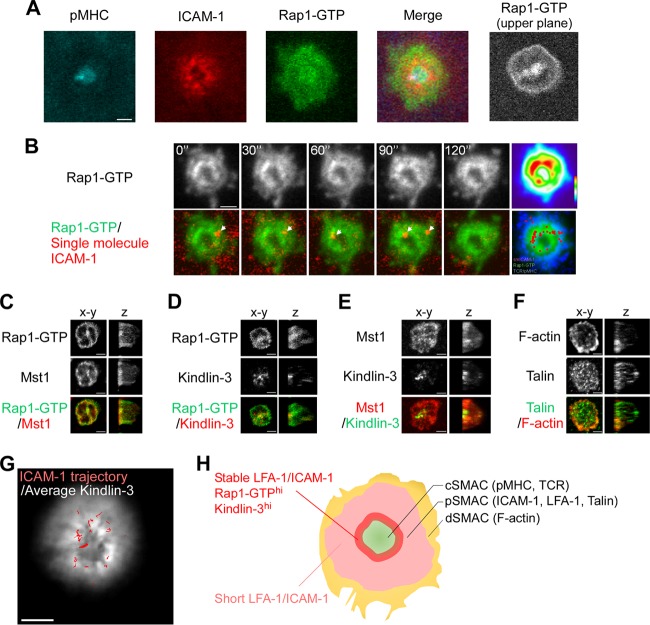
Distribution of activated Rap1 and high-affinity ICAM-1 binding at the inner zone of the pSMAC. (A) Localization of Rap1-GTP. Representative images of IS in naive OT-II T cells expressing the Rap affinity probe. TIRF images of pMHC, ICAM-1, and Rap1-GTP and a merged image. Confocal image of Rap1-GTP at 0.5 μm above the contact plane (upper plane). (B) Time-lapse images of stable ICAM-1 binding and Rap1-GTP in IS of naive OT-II T cells expressing the Rap affinity probe. Sequential images with a 30-s interval of Rap1-GTP (top) and merged images of ICAM-1 spots (bottom) are shown. ICAM-1 spots (red) are indicated with arrows. (Upper) Heat map presentation of the averaged images of Rap1-GTP. (Lower) Distribution of stable ICAM-1 binding spots (red) overlaid on the averaged image of Rap1-GTP (green) and pMHC microcluster (blue). (C to F) OT-II T cells expressing the Rap affinity probe were subjected to immunostaining for the expression of Mst1 (C), kindlin-3 (D), kindlin-3/Mst1 (E), and talin/F-actin (F). Front and side views of reconstructed 3D confocal images are shown. (G) IS of an OT-II T cell blast expressing EGFP–kindlin-3. Cultured OT-II T cell blasts expressing EGFP–kindlin-3 were time-lapse imaged on lipid bilayers as described for panel B. An averaged image of EGFP–kindlin-3 overlaid with stable ICAM-1 bindings (red) is shown. Note that EGFP–kindlin-3 was accumulated at the inner pSMAC zone, where stable ICAM-1 bindings frequently occurred. Scale bar, 2.5 μm. (H) Schematic summary of Rap1 activation patterns, kindlin-3 localization, and LFA-1/ICAM-1 interaction in IS.

TIRF microcopy was used to examine spatiotemporal patterns of Rap1 activation. We detected characteristic patterns of activated Rap1 distribution in the pSMAC with intense accumulation at the inner zones of the pSMAC region (Fig. 3A). Confocal images above the contact plane showed activated Rap1 accumulation at the plasma membrane as well as the restricted areas above the cSMAC, presumably near the microtubule-organizing center (Fig. 3A and Fig. S3B). Although time-lapse imaging revealed dynamic changes in the distribution of activated Rap1 at the periphery of the contact membrane, the inner Rap1 activation zone was relatively stable over time (Fig. 3B, upper). Time-lapse microscopy that captured 10-s-interval images revealed only stable ICAM-1 binding. Multicolor time-lapse imaging of the Rap affinity probe revealed stable ICAM-1 arrest that frequently occurred in sites of high Rap1 activation near cSMAC, in contrast to peripheral occurrence of pMHC microclusters (Fig. 3B, lower, and Videos S4 and S5), suggesting that sustained Rap1 activation is required for high-affinity binding to ICAM-1.

We fixed OT-II T cells bearing the Rap affinity probe and immunostained for Mst1, kindlin-3, and talin to examine their three-dimensional (3D) distribution using reconstituted confocal images. In mature synapses, Mst1 accumulated and colocalized with activated Rap1 at the contact membrane and inner pSMAC region. Mst1 and Rap1-GTP were also colocalized outside pSMAC (dSMAC) and were not visualized by TIRF microscopy (Fig. 3C). Kindlin-3 was located in the pSMAC with higher intensities in the inner pSMAC areas (Fig. 3D and E); kindlin-3 immunostaining was abolished by the addition of glutathione *S*-transferase–kindlin-3, confirming the specific staining (Fig. S3C). talin was broadly distributed as punctae throughout the pSMAC, and F-actin developed in dSMAC with small punctae in the pSMAC (Fig. 3F). Thus, kindlin-3 is preferentially localized to the inner pSMAC zone enriched for Rap1-GTP.

We next examined the spatial relationship between stable LFA-1/ICAM-1 binding events and kindlin-3 in the IS of living T cells. Enhanced GFP (EGFP)–kindlin-3 was expressed in OT-II T cell blasts, and ICAM-1 binding was measured on supported bilayers. EGFP–kindlin-3 was distributed in the contact area and accumulated near the cSMAC (Fig. 3G). Time-lapse imaging identified high-affinity ICAM-1 binding lasting longer than 10 s in kindlin-3-rich regions (Fig. 3G and Video S6). Collectively, these findings revealed an inner pSMAC zone characterized by large amounts of activated Rap1 and kindlin-3 in which stable ICAM-1 binding events frequently occurred (Fig. 3H).

### Rap1 signaling is required for kindlin-3 recruitment to support high-affinity binding to ICAM-1.

**FIG 4:**
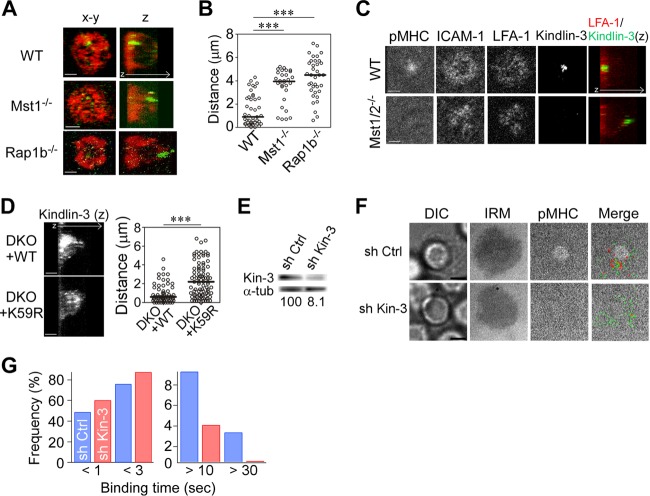
Recruitment of kindlin-3 is required for high-affinity ICAM-1 binding. (A) Front and side 3D views showing the distribution of talin (red) and kindlin-3 (green) in IS of wild-type (WT), *Mst1*^−/−^, and *Rap1b*^−/−^ OT-II T cells. (B) The distance of the peak intensity of kindlin-3 from the contact plane. ***, *P* < 0.001. (C) Confocal images of pMHC, ICAM-1, LFA-1, and kindlin-3 at the contact plane as well as side 3D views of kindlin-3 (green)/LFA-1 (red) in wild-type (upper) and *Mst1*^−/−^/*Mst2*^−/−^ (lower) T cells. (D) The distance of peak intensity in *Mst1*^−/−^/*Mst2*^−/−^ OT-II T cell blasts expressing wild-type Mst1 (DKO+WT) or kinase-inactive Mst1 mutant (K59R) (DKO+K59R). ***, *P* < 0.001. (E to G) Single-molecule measurement of LFA-1/ICAM-1 binding events using kindlin-3-depleted OT-II T cell blasts. (E) Knockdown of kindlin-3 in OT-II T cell blasts. Expression of kindlin-3 in cultured OT-II T cells transduced with control shRNA (sh Ctrl) and kindlin-3-specific shRNA (sh Kin-3). (Bottom) Shown are the percentages of kindlin-3 expression normalized to α-tubulin expression. (F) Representative images of IS in control and kindlin-3 knockdown T cell blasts. DIC, IRM, cSMAC (pMHC), and merged images of cSMAC with trajectories of LFA-1/ICAM-1 bindings in control (sh Ctrl) and kindlin-3 knockdown (sh Kin-3) OT-II T cell blasts are shown. (G) LFA-1/ICAM-1 binding lifetime in IS of control (blue) and kindlin-3 knockdown (red) cells calculated from 828 and 831 trajectories, respectively. Scale bar, 2.5 μm. The two-way chi-square *P* value was <0.0001.

To explore whether an impairment of Rap1 signaling would impact talin and kindlin-3 distribution, IS of naive *Mst1*^−/−^ or *Rap1b*^−/−^ OT-II T cells were immunostained and examined for talin and kindlin-3 localization in reconstructed 3D images. As shown before, talin was accumulated and widely localized in pSMAC in wild-type T cells, while kindlin-3 was localized to the inner pSMAC zone (Fig. 4A). In Mst1-deficient T cells, talin was present at the contact membrane, although its distribution was less concentrated than that in wild-type cells. However, kindlin-3 was largely localized to the opposite side away from the contact surface. Similar defects were found in the IS of *Rap1b*^−/−^ T cells (Fig. 4A). The distance of the peak intensities of kindlin-3 in *Mst1*^−/−^ or *Rap1b*^−/−^ T cells from the contact surface was significantly greater than that of wild-type cells (Fig. 4B). Knockdown of RAPL also resulted in mislocalized kindlin-3 (Fig. S4A and B). LFA-1 normally accumulated at the contact site and localized with ICAM-1 in wild-type T cells (Fig. 4C). On the other hand, Mst1/2 DKO T cells exhibited reduced peak intensities of LFA-1 and mislocalized kindlin-3 (Fig. 4C). It is important to note that normal localization of kindlin-3 was restored following lentiviral transfer of wild-type Mst1, but not the inactive kinase mutant (K59R), into Mst1/2 DKO cells (Fig. 4D). Therefore, Mst1 kinase activity is necessary to induce proper localization of kindlin-3.

The strong accumulation of kindlin-3 near the cSMAC and its mislocalization in the IS of *Rap1b*^−/−^ and *Mst1*^−/−^ T cells suggest that kindlin-3 is critical for high-affinity ICAM-1 binding. To examine whether kindlin-3 is required for high-affinity ICAM-1 binding, kindlin-3 was silenced using lentiviral transfer of shRNA specific for kindlin-3 into OT-II T cell blasts. Kindlin-3 protein expression was reduced by more than 90% (Fig. 4E). Compared to control T cell blasts, kindlin-3 knockdown T cell blasts had less stable ICAM-1 binding and severely diminished cSMAC formation (Fig. 4F). Moreover, kindlin-3 depletion significantly decreased ICAM-1 binding events longer than 10 s with a reciprocal increase of short 3-s binding events (*P* < 0.0001) (Fig. 4G). These data clearly indicate that kindlin-3 is critically important for high-affinity interactions between LFA-1 and ICAM-1 and for cSMAC development.

### Mislocalization of vesicle transport regulators in IS by defective Rap1 signaling.

**FIG 5:**
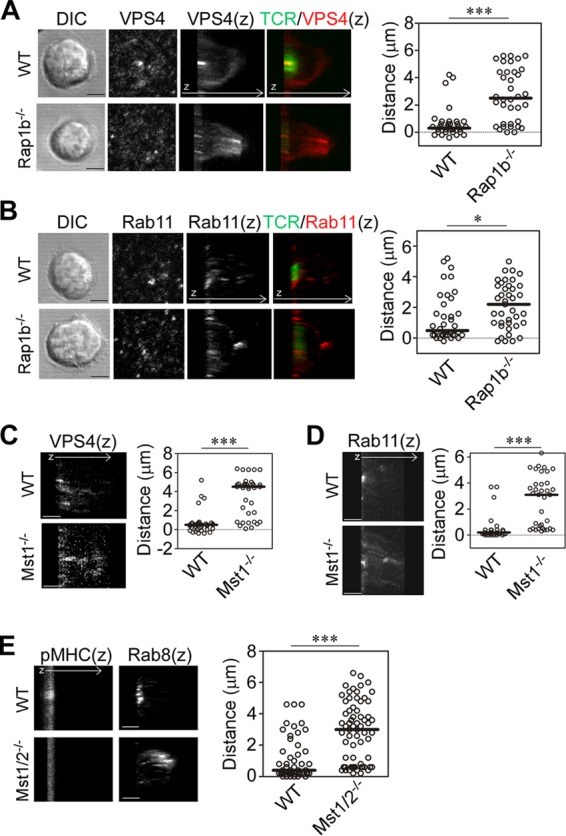
Mislocalized vesicle transport regulators in IS of *Rap1b*^−/−^ or *Mst1*^−/−^ T cells. (A and B) Localization of VPS4 and Rab11 in IS of naive OT-II T cells from wild-type and *Rap1b*^−/−^ mice. Shown are DIC and images of contact plane and 3D images (*z*) of TCR and VPS4 (A) or Rab11 (B). (C and D) 3D images (*z*) of VPS4 (C) and Rab11 (D) in *Mst1*^−/−^ naive OT-II T cells. (E) 3D images (*z*) of Rab8 and pMHC in naive *Mst1*^−/−^/*Mst2*^−/−^ (Mst1/2^−/−^) OT-II T cells. ***, *P* < 0.001; *, *P* < 0.05. Scale bar, 2.5 μm. Distances of the peak intensities from the contact plane were quantified and are shown on the right.

Polarized recycling and release of microvesicles containing TCR contribute to cSMAC formation (11, 12). The impairment of cSMAC formation in Rap1- or Mst1/Mst2-deficient T cells suggests that defective vesicle transport is involved. To clarify this, we examined the localization of the ESCRT component vacuolar protein sorting 4 (VPS4), which acts at the scission step of TCR-enriched microvesicles (12). As previously reported (12), VPS4 primarily accumulated in the TCR-enriched regions of IS in naive OT-II T cells (Fig. 5A). In contrast, VPS4 did not accumulate at IS of *Rap1b*^−/−^ T cells and was instead distributed away from the contact surface. VPS4 was within 0.6 μm (median, 0.3 μm) of the contact surface in wild-type T cells, but it was significantly further away in *Rap1b*^−/−^ T cells (*P* < 0.001) (Fig. 5A). TCR/CD3 complexes are recycled to the IS by vesicle transport components, including Rab11 and Rab8 (10, 11). In wild-type cells, Rab11 localized to the cSMAC within an average distance of 1.4 μm (median, 0.5 μm), whereas in *Rap1b*^−/−^ T cells Rab11 was broadly distributed away from the contact surface within 2.1 μm (median, 2.2 μm) (Fig. 5B). Similarly, VPS4, Rab11, and Rab8 were abnormally distributed in Mst1 KO T cells and Mst1/2 DKO T cells (Fig. 5C to E).

We hypothesized that high-affinity binding of ICAM-1 controls localization of the recycling and release machinery of the TCR complex. To examine this possibility, Mn^2+^ was used in combination with pMHC. Indeed, Mn^2+^ increased the localization of VPS4 toward the cSMAC with augmented cSMAC and pSMAC formation in *Rap1b*^−/−^ T cells (Fig. S5A and B), suggesting that high-affinity ICAM-1 binding facilitates IS formation. However, mature IS with clear cSMAC and pSMAC was not recovered in Rap1 DKO T cells under the same conditions (Fig. S5C), highlighting an essential role for Rap1 in the stabilization of high-affinity LFA-1 binding to ICAM-1 and cSMAC formation.

### Downstream Mst1/2 signaling via NDR1 kinase plays an important role in SMAC maturation.

**FIG 6:**
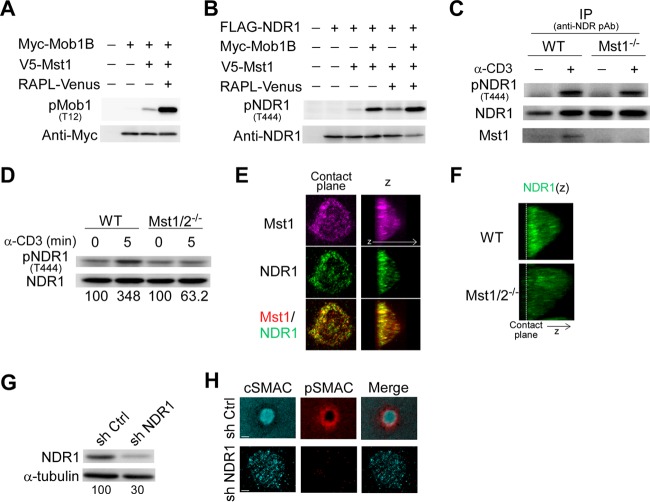
Requirement of TCR-triggered activation of NDR1 for IS formation. (A) Enhanced phosphorylation of Mob1B by Mst1 in the presence of RAPL. Mob1B phosphorylation was detected with anti-phospho-Mob1B (pT12) following transfection with Myc-Mob1B, V5-Mst1, and RAPL-Venus as indicated. (B) RAPL and Mob1B induced NDR1 phosphorylation by Mst1. Phosphorylation of NDR1 (pT444) was detected with anti-phospho-NDR antibody (pAb). (C) Phosphorylation of NDR1 (pT444) in wild-type and *Mst1*^−/−^ T cells after TCR ligation. T cells stimulated with anti-CD3 or left unstimulated were examined for NDR1 phosphorylation after immunoprecipitation (IP) of NDR1. The blots were reprobed for NDR1 and Mst1. (D) Phosphorylation of NDR1 (pT444) in wild-type and Mst1/2 DKO T cells were examined as described above. Percentages of NDR1 phosphorylation were normalized to total NDR1. (E) Localization of Mst1 and NDR1 in IS. OT-II T cell IS were fixed and immunostained for Mst1 (magenta) and NDR1 (green). Colocalization is indicated by yellow in the merged image (bottom). (F) 3D image (*z*) for NDR1 in IS of OT-II T cell blasts from wild-type and Mst1/2 DKO mice. The dashed line indicates the contact plane. (G) Knockdown of NDR1 with shRNA. Expression of NDR1 in cultured OT-II T cells transduced with lentiviral vectors containing control shRNA (sh Ctrl) and NDR1-specific shRNA (sh NDR1). Values at the bottom indicate the percentages of NDR1 expression normalized to α-tubulin expression. (H) Representative images of IS (cSMAC, pSMAC, and merged) in NDR1 knockdown T cell blasts. Scale bars, 2.5 μm.

NDR kinases are activated through phosphorylation at the C-terminal hydrophobic motif by external kinases such as Mst1 and Mst3 kinases (48, 49). Chemokine-triggered activation of NDR1/2 occurs in an Mst1-dependent manner in T cells and plays an important role in T cell trafficking (37). Understanding that Rap1 signaling to Mst1/2 was required for IS formation, we hypothesized that NDR1, the major isoform expressed in T cells (37), acts downstream of Mst1/2 and contributes to IS formation. We first examined whether Mst1 and RAPL could stimulate NDR1 phosphorylation using 293T cells. Mst1 modestly phosphorylated Mob1B, a coactivator of NDR kinases (48), whereas RAPL markedly augmented Mob1B phosphorylation by Mst1, confirming that RAPL can efficiently activate Mst1 kinase activities (Fig. 6A). Mst1 alone only marginally phosphorylated the carboxyl-terminal T444 residue of NDR1. In contrast, phosphorylation of NDR1 was increased in the presence of Mob1B or RAPL and was further augmented with coexpression of Mob1B and RAPL (Fig. 6B). In primary T cells, we detected basal levels of NDR1 phosphorylation. When stimulated with an activating anti-CD3 monoclonal Ab, NDR1 was further phosphorylated and immunoprecipitated with Mst1 (Fig. 6C). NDR1 phosphorylation was modestly attenuated in Mst1-deficient T cells (Fig. 6C) and largely abolished in Mst1/2 DKO T cells (Fig. 6D). Thus, NDR1 activation occurred in an Mst1/2-dependent manner following TCR triggering.

In support of these findings, we found that NDR1 accumulated at contact membranes and colocalized with Mst1 in IS formed by OT-II T cells (Fig. 6E). However, in Mst1/2 DKO T cells NDR1 was diffusely distributed throughout the cytoplasm (Fig. 6F). To identify the role of NDR1 kinase, shRNA-mediated knockdown decreased NDR1 protein levels by 70% (Fig. 6G) and exhibited an impairment of mature IS formation. Central clustering of both pMHC and ICAM-1 ring was not clearly formed (Fig. 6H). Collectively, these results indicate that TCR-triggered activation of NDR1 is mediated by Mst1/Mst2 and is required for IS maturation.

### NDR1 regulates kindlin-3 localization and IS formation.

**FIG 7:**
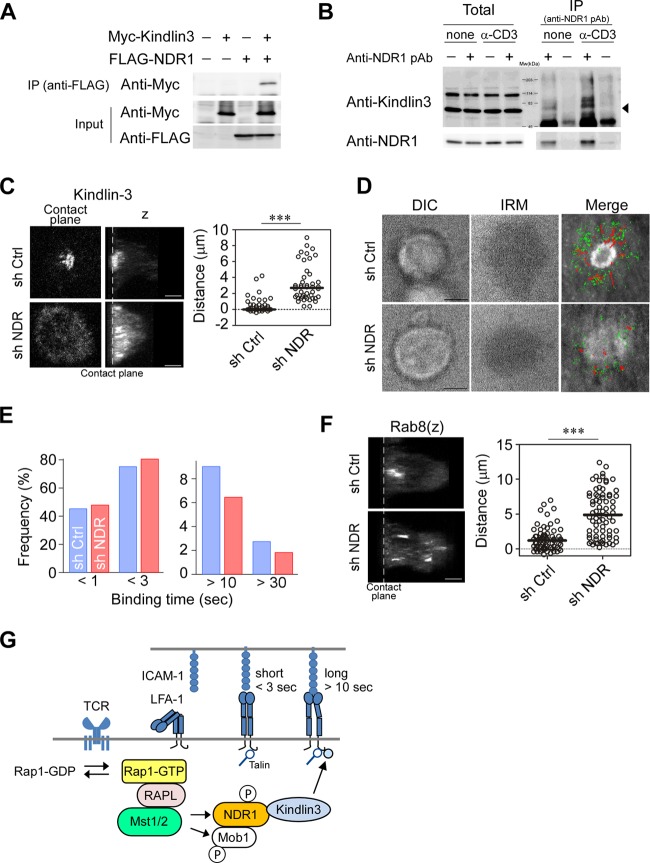
NDR1 regulates localization of kindlin-3 and Rab8. (A) Association of NDR1 and kindlin-3. 293T cells transfected with Myc–kindlin-3 and FLAG-NDR1 as indicated were subjected to immunoprecipitation (IP) of FLAG-NDR1 followed by immunoblotting for Myc–kindlin-3. (B) Primary T cells stimulated with anti-CD3 or left unstimulated were analyzed for association of NDR1 with kindlin-3. IP with anti-NDR Ab was immunoblotted for kindlin-3. Total and precipitated proteins are indicated. The arrowhead indicates the predicted molecular mass of kindlin-3 (75.6 kDa). (C) Mislocalized kindlin-3 in NDR1 knockdown T cell blasts. Representative views at the contact plane and 3D images (*z*) of NDR1 knockdown (sh NDR) and control (sh Ctrl) OT-II T cells. Distances of peak intensities from the contact plane of kindlin-3 are shown. Scale bars, 2.5 μm. ***, *P* < 0.001. (D and E) Single-molecule measurement of LFA-1–ICAM-1 interactions for control and NDR1 knockdown T cell blasts. (D) Representative images of DIC, IRM, and the cSMAC overlaid with trajectories of LFA-1/ICAM-1 binding in control and NDR1 knockdown OT-II T cell blasts. (E) Profiles of ICAM-1 binding lifetime in control (blue) and NDR1 knockdown (red) cells were calculated from 1,568 and 1,993 trajectories, respectively. The two-way chi-square *P* value was 0.003. (F) 3D images (*z*) of Rab8 in IS of control and NDR1 knockdown OT-II T cells. (Right) Distances of peak intensities from the contact plane are shown. Scale bar, 2.5 μm. ***, *P* < 0.001. (G) A schema of the Rap1 signaling pathway to kindlin-3.

To explore whether NDR1 regulates kindlin-3, the interaction of NDR1 and kindlin-3 was examined using 293T cells. We found that kindlin-3 coimmunoprecipitated with NDR1 (Fig. 7A) and vice versa (Fig. S6A). To confirm whether the association of NDR1 and kindlin-3 also occurs in T cells, T cells were stimulated with anti-CD3 and subjected to immunoprecipitation. We found that kindlin-3 coimmunoprecipitated with NDR1 in the presence or absence of anti-CD3 stimulation (Fig. 7B). These findings prompted us to examine the effects of NDR1 on phosphorylation and localization of kindlin-3. We immunoprecipitated activated NDR1, which was subjected to an *in vitro* kinase assay using recombinant kindlin-3 or kinase peptide substrate as a control. While activated NDR1 phosphorylated the peptide substrate, we did not detect phosphorylation of kindlin-3 (Fig. S6B and C). To clarify the effect on kindlin-3 localization, we depleted NDR1 by using shRNA and immunostained OT-II IS for kindlin-3. While control OT-II T cell blasts exhibited localized kindlin-3 near cSMAC as shown before, NDR1 knockdown resulted in defective cSMAC generation with diffuse distribution of kindlin-3, which was significantly distributed away from the contact surface (Fig. 7C and D). Overexpression of a kinase-deficient mutant, NDR1 (D212A), in cultured OT-II T cells reduced attachment and defective accumulation of kindlin-3 (Fig. S6D), suggesting that the kinase activity of NDR1 is required. In agreement with perturbed kindlin-3 localization, the single-molecule analysis of ICAM-1 on lipid bilayers revealed that high-affinity ICAM-1 binding events were significantly reduced in NDR1 knockdown OT-II T cell blasts (*P* = 0.003) (Fig. 7E). Moreover, high-affinity ICAM-1 binding failed to accumulate in the inner pSMAC region and did not exhibit the apparent centralized movements seen in the control (Fig. 7D). Similar to our findings in Mst1/2 DKO T cells, NDR1 knockdown also resulted in dispersed Rab8 localization (Fig. 7F). Collectively, these results suggest that NDR1 acts downstream of Mst1/2 to regulate the localization of kindlin-3 for high-affinity binding to ICAM-1 and cSMAC formation (Fig. 7G).

## DISCUSSION

Here, we established a quantitative method to measure the dynamics of ICAM-1 binding at the single-molecule level in IS of naive T cells on supported lipid bilayers presenting agonist pMHC. Stable binding between LFA-1 and ICAM-1, corresponding to high-affinity interactions, preferentially occurred at the activated Rap1-enriched inner pSMAC zone. Although high-affinity interactions occurred at low frequencies, our findings indicate that they are essential for mature IS formation. Kindlin-3 was localized to the inner Rap1-activated zone, which was relatively stable over time and space, suggesting a platform for sustained Rap1 signaling to kindlin-3 for the maintenance of stable binding. Indeed, Rap1 signaling to kindlin-3 was required for stabilization of LFA-1/ICAM-1 binding, which is in agreement with the role of kindlin-3 in postadhesion events (22, 23). In line with this notion, the inhibition of Rap1 signaling led to breakdown of IS formation in mature thymocytes (46). The observation that LFA-1 activation by Mn^2+^ failed to rescue IS formation in Rap1-deficient T cells further highlights the requirements for the formation of Rap1 signalosome at the inner pSMAC region to sustain LFA-1 binding to ICAM-1 and mature IS formation.

Although kindlin-3 is critically required for integrin-dependent adhesion of leukocytes and platelets, our understanding of the molecular mechanisms of kindlin-3 regulation remains limited. We showed that Mst1/Mst2 played a critical role in the recruitment of kindlin-3 to the inner pSMAC zone. Our study identified NDR1 kinase as a key signaling molecule downstream of Mst1/Mst2. Biochemically, activation of NDR kinases requires the phosphorylation of the C-terminal hydrophobic motif (pT442/444) and Mob1B as a coactivator (48, 49). NDR1 and Mob1B were phosphorylated by Mst1 in the presence of a Rap1-binding protein, RAPL. Upon TCR stimulation, NDR1 was phosphorylated in an Mst1/Mst2-dependent manner, indicating that Mst1/Mst2 mediates TCR-triggered NDR1 activation. Importantly, NDR1 associated with kindlin-3. Although NDR1 did not directly phosphorylate kindlin-3, the results of NDR1 knockdown and overexpression of kinase-deficient NDR1 suggest that the NDR1 kinase activity requires kindlin-3 recruitment to the IS. The Rap1 signaling pathway to kindlin-3 revealed in this study is schematically summarized in Fig. 7G.

In addition to defects in stable binding to ICAM-1, impairments of Rap1, Mst1/Mst2, and NDR1 accompanied mislocalized vesicle transport regulators/releasing machineries that are important to the formation of the cSMAC, suggesting a central role of NDR1 in recruitment of kindlin-3 and TCR recycling/releasing through its activation of polarized vesicle transports. It is important to note that the linkage of LFA-1/ICAM-1 binding with cSMAC formation has been reported in cytotoxic immune synapse formation in NK cells, where LFA-1/ICAM-1 binding was required for the formation of the central zone of the synapse and confined release of lytic enzymes (50). A recent study using high-resolution live imaging of the cytotoxic T lymphocyte (CTL) IS detected a coordinated polarization of the centrosome and vesicular traffic for the formation of the cSMAC (51). Furthermore, stable LFA-1/ICAM-1 binding may provide a positional cue for reorganization of the centrosome and vesicle transport. Interestingly, intraflagellar transport machinery components interplay with the Rab GTPase network (Rab5, Rab8, and Rab11) and play a crucial role in TCR recycling to the IS (10). NDR1/2 kinases play important roles in ciliary transport (52) and spine formation (53) through the Rab8 exchange factor Rabin8, β1-integrin activation and trafficking, and integrin-dependent neurite growth (54). Mst1/Mst2 was implicated in ciliogenesis by promoting ciliary localization of multiple ciliary cargoes (55). Taken together, these studies suggest that Rap1 signaling to NDR kinases orchestrates vesicular transport of TCR and adhesion machineries. Rap1/RAPL/Mst1 also associates with the same vesicle compartments as LFA-1 (27), suggesting that polarized vesicular transport to IS is key to sustained, stable LFA-1/ICAM-1 binding. Thus, it is conceivable that the Rap1 signalosome is generated and maintained through a positive-feedback loop through sustained LFA-1/ICAM-1 binding that reorients vesicle transport to supply Rap1 signaling components and LFA-1 to IS. In line with this notion, defective Rap1 signaling resulted in diminished LFA-1 and mislocalized kindlin-3 in IS. Further studies are needed to dissect the requirements for coordinated vesicle transport systems to generate the Rap1 signalosome and its relationship with TCR recycling/releasing processes.

Although high-affinity LFA-1 conformations have been described in numerous studies, there is limited information regarding high-affinity interactions with TCR ligation in ligand binding assays using soluble ICAM-1 (56), suggesting that events after ligand binding to immobilized ligand are necessary for induction of high-affinity integrins (20). Indeed, our study showed that compared to transient low/intermediate-affinity binding events, high-affinity LFA-1/ICAM-1 binding events in naive T cells exhibited low diffusion coefficients on planar lipid bilayers, suggesting that bound LFA-1 at the inner pSMAC is firmly anchored to cytoskeletal structures. Comrie et al. reported concentric distribution of high-affinity conformations of LFA-1 near cSMAC in primary human T cells on planar bilayers presenting ICAM-1 and anti-CD3 antibodies (38). Their study proposed a model with an important role of F-actin retrograde flow for generation of high-affinity conformers through the effect of lateral force on F-actin-anchored engaged LFA-1. Although distribution of high-affinity conformers near cSMAC is in agreement with our study, they did not demonstrate that LFA-1 movement along F-actin retrograde flow accompanies affinity maturation. In addition, a caveat of that study is that the use of m24, which stabilizes high-affinity LFA-1 (57), could augment high-affinity conformations and LFA-1 clustering on the membrane. The SPT analysis in our study showed that kinetic profiles of LFA-1/ICAM-1 bindings in naive and activated T cell blasts were comparable, while ICAM-1 centripetal movements were rarely seen in naive T cells, suggesting that centripetal movements of LFA-1/ICAM-1 complexes are a unique property in F-actin-rich, activated T cell blasts and the like and are not essential for high-affinity induction. Our study suggests that a restraint on movement of engaged LFA-1 anchored to cytoskeletal structures would provide a mechanism to promote conformational changes.

The observation of the broad distribution of talin puncta and concentric kindlin-3 localization in the pSMAC, together with LFA-1/ICAM-1 binding patterns in IS, suggests distinct roles of talin and kindlin-3 in LFA-1 regulation. Talin was required for the induction of extended, intermediate LFA-1 conformers. On the other hand, kindlin-3 was required for high-affinity conformations and stabilization of LFA-1/ICAM-1 bindings (22, 23, 58). It is currently unknown whether talin and kindlin-3 act sequentially or simultaneously to induce high-affinity LFA-1 binding. Since the inner pSMAC regions are relatively poor in F-actin, kindlin-3 may stabilize talin binding to integrins and F-actin. Alternatively, kindlin-3 could independently contribute to anchoring. For example, a close correlation was detected between sites where microtubules and LFA-1 are clustered in IS of CTLs (59). It is possible that the association between RAPL and NDR1 with microtubules (60, 61) contributes to the stabilization of LFA-1/ICAM-1 to microtubules.

Here, we demonstrated that the Rap1-RAPL-Mst1/2-NDR1 signaling axis plays crucial roles during SMAC formation via the establishment of high-affinity LFA-1/ICAM-1 binding at the inner pSMAC through kindlin-3 and targeting of the TCR recycling/releasing machineries to IS. Our findings support the notion that the generation and clearance of the Rap1 signalosome at the inner pSMAC zone controls IS stability and phased T cell adhesion behaviors during T-APC interactions *in vivo*, thus influencing effector/memory functions of T cells. Moreover, the tools and insights described in this study will be helpful as we conduct further studies to dissect the molecular mechanisms underlying T cell activation and the acquisition of effector/memory functions.

## MATERIALS AND METHODS

### Antibodies and reagents.

Anti-kindlin-3 Ab and anti-Mst1 Ab, used for immunoblotting, were purchased from Millipore. Anti-Mst1 Ab, phalloidin-iFluor 647, anti-NDR1 Ab, and anti-kindlin-3 Ab, used for immunoblotting, were purchased from Abcam. Antitalin Ab and anti-FLAG Ab were purchased from Sigma-Aldrich. Anti-VPS4 was purchased from Santa Cruz Biotechnology. Anti-Rab11 and anti-Rap1 Abs were purchased from BD Transduction Laboratories. Anti-Rab8 Ab and anti-pMob1B Ab were purchased from Cell Signaling Technologies. Affinity-purified anti-pNDR (T442/444) (MBL) was generated as described previously (62). Alexa Fluor 488-, 555-, or 633-conjugated anti-rabbit, -mouse, and -rat IgG Abs, as well as Alexa Fluor 488-conjugated streptavidin (StAv), were purchased from Invitrogen. Anti-LFA-1 (β2) was purchased from Bioworld Technology. Anti-TCRβ–fluorescein isothiocyanate (FITC) Ab was purchased from eBioscience. Anti-Myc (9E10) was purified from a hybridoma purchased from ATCC. ATTO425- or ATTO565-conjugated StAv and ATTO 647N–*N*-hydroxysuccinimide (NHS) ester were purchased from ATTO-TEC. CF568-NHS ester was purchased from Biotium. Monobiotinylated I-Ab with 0.1 M OVA peptide (AAHAEINEA) was purchased from the NIH Tetramer Core Facility.

### Mice.

Mice with *loxP*-flanked alleles encoding *Rap1b* were generated using a standard gene-targeting method for C57BL/6 embryonic stem cells. *Rap1a*-null/*Rap1b*^*fl/fl*^/*CD4-Cre* mice, *Mst1*^*fl/fl*^ mice, and *Mst2*-null mice on the C57BL/6 background were generated as previously described (28, 46, 63). *OT-II* mice expressing a TCR specific for OVA_323–339_ bound to I-A^b^, *αL*-null mice, and *β2*-null mice were obtained from Jackson Laboratories. C57BL/6 mice were purchased from CLEA Japan. *OT-II*/*Rap1b*^*fl/fl*^/*CD4-Cre* mice and *OT-II*/*Mst1*^*fl/fl*^/*CD4-Cre*/*Mst2*-null mice were generated by intercrossing. Littermates were used as controls. All mice were maintained under specific-pathogen-free conditions in the animal facility at Kansai Medical University (Osaka, Japan) and were treated and used for experiments in accordance with institutional guidelines and ethical approvals for the care of experimental animals.

### Lipid bilayers.

Mouse ICAM-1-GPI was purified and fluorescently labeled with CF568-NHS-ester (Biotium) and incorporated into liposomes consisting of 0.4 mM 1,2-dioleoyl-*sn*-glycero-3-phosphocholine (DOPC) containing 0.1 mol% biotin-CAP-phycoerythrin (PE) (Avanti Polar Lipids, Alabaster, AL) essentially as described previously (32, 64). For single-molecule imaging, ICAM-1 was labeled with ATTO-647N-NHS-ester (50 pmol) and titrated for single-molecule imaging conditions. ICAM-1 liposomes were mixed with DOPC/CAP-biotin-PE liposomes (final concentrations of 0.4 mM DOPC and 0.1 mol% biotin-CAP-PE) and deposited onto piranha-treated cover glasses mounted onto a 35-mm dish. Lipid bilayers were blocked with 1% bovine serum albumin (BSA) and loaded sequentially with 2 μg/ml streptavidin-conjugated Alexa Fluor 488 or ATTO425, followed by monobiotinylated I-A^b^ coupled with OVA peptide (AAHAEINEA). The site densities were determined on silica beads using anti-ICAM-1 (YN1.1) and anti-MHC class II (M5/114.15.2) as previously described (64). To support IS formation of naive OT-II T cells, we used site densities of 300 molecules/μm^2^ and 200 molecules/μm^2^ for ICAM-1 and pMHC, respectively.

### Immunological synapse.

For live-cell imaging, 5 × 10^5^ freshly isolated naive T cells (CD62L^hi^ CD44^lo^ CD4^+^) from OT-II wild-type and mutant mice were suspended in phenol red-free RPMI supplemented with 4% fetal calf serum (FCS) and 10 mM HEPES (pH 7.5) before being applied onto lipid bilayers presenting ICAM-1 and pMHC as described above (32, 46). The cells were incubated on the lipid bilayers, and single-molecule video rate imaging was performed at a 33-ms interval using an iXonUltra electron-multiplying charge-coupled-device camera (Andor) fitted with a 100× objective lens (numeric aperture [NA], 1.45) and Solis software. The photostability of ATTO647N-labeled ICAM-1 was a *t*_1/2_ of approximately 3 min under our imaging conditions (see Fig. S1B in the supplemental material). We captured ICAM-1 images for 200 s and images of differential interference contrast microscopy (DIC), interference reflection microscopy (IRM), and cSMAC before and after video rate ICAM-1 imaging.

### Single-particle tracking and data analysis.

In cropped areas containing single cells, ICAM-1 spots were automatically captured for the central peak coordinates with Gaussian fitting after filtering the intensity and size of spots and tracked using G-count software (G-Angstrom, Japan) with a defined tracking radius (see details in the supplemental material), and trajectories were analyzed using G-track software to exclude erroneous tracks. For further track analyses, data sets (*x*, *y*, and *t*) of each track were transferred to MATLAB software to plot with cSMAC or IRM images and to calculate mean squared displacements (MSD). The diffusion coefficients were calculated from the slope of the MSD curve over time fitted with the least-squares method using the formula <*x*^2^> = 4*Dt*, where *D* represents the diffusion coefficient (65). Individual ICAM-1 spot diffusion in cell-free areas was in agreement with membrane proteins (0.1 to 0.5 μm^2^/s) and consistent with single-molecule features, including single peaks, spot diameters equivalent to diffraction limits, and single-step photobleaching (Fig. S1C and D).

The SPT with a 0.22-μm search radius eliminated freely mobile ICAM-1 and detected slow-moving ICAM-1 spots in wild-type T cells, while OT-II T cells derived from αL- or β2-deficient mice rarely exhibited detectable ICAM-1 spots (Fig. S1E and F). The antibodies for LFA-1 and ICAM-1 also severely diminished ICAM-1 spots under the same conditions. The lower limit of detection for arrest events by SPT was 0.5 s using a 99% cutoff value. We analyzed tracks longer than 0.5 s in order to obtain at least 15 positions. The upper limit of our SPT analysis to detect binding events was set at 200 s based on the photostability of the dye under our imaging laser power (*t*_1/2_ of 3 min). The time range (0.5 to 200 s) covers average binding lifetimes deduced from *k*_off_ rates (a reciprocal of *k*_off_) of recombinant conformation-locked low (0.2 to 0.8 s)-, intermediate (1.3 to 2.3 s)-, or high (22 to 71 s)-affinity I domains (40) or intact high-affinity LFA-1 (33 s) (41). In this study, we set 10 s or longer for high-affinity binding interactions.

### Antigen-dependent proliferation of T cells.

OT-II T cells from wild-type and mutant mice were labeled with 3 μM 5-(and 6)-carboxyfluorescein diacetate succinimidyl ester (CFSE). Fifty thousand T cells were cocultured for 3 days with bone marrow-derived dendritic cells (10^4^ cells) in the presence of various concentrations of OVA_323–339_ peptides in a 96-well flat-bottom plate. Fractions of proliferating cells were measured by CFSE dilution (66) after being stained with anti-Vβ5-PE and anti-CD4-APC for OT-II T cells.

### Immunoprecipitation assays.

Immunoprecipitation and immunoblotting were performed as previously described (27, 46). HEK293T cells were transfected with pcDNA3.1 (Invitrogen) encoding Myc-Mob1B, V5-Mst1, RAPL-Venus, or FLAG-NDR1 using Fugene 6 reagent (Promega). Cells were lysed with lysis buffer (1% NP-40, 20% glycerol, 150 mM NaCl, 25 mM Tris, pH 7.5, 2 mM MgCl_2_, 1 mM phenylmethylsulfonyl fluoride [PMSF], 50 mM NaF, 2.5 mM NaVO_4_, 20 mM disodium β-glycerophosphate, 5 μg/ml leupeptin) for 20 min on ice, followed by centrifugation for 10 min at 4°C. Naive T cells were isolated from the spleen and lymph nodes of wild-type and mutant mice using a Pan T cell isolation kit II (Miltenyi Biotec) and were stimulated with or without anti-CD3ε (2C11; 10 μg) for 5 min. Whole-cell lysates were harvested from the cells and subjected to immunoprecipitation with tag antibodies for transfection experiments or anti-NDR1 polyclonal antibody (Abcam) for primary T cells, followed by incubation with protein G-Sepharose (GE Healthcare). After washing with lysis buffer four times, immunoprecipitates were subjected to immunoblotting.

### Lentivirus transfer of shRNA and cDNA.

Target sequences (19 nucleotides) for shRNA knockdown were selected using online public software, and their knockdown efficiencies were examined using lymphoid cell lines. Control sequences and target sequences with high efficiencies were expressed in a lentivirus vector with an H1 promoter (provided by H. Miyoshi, RIKEN, Japan) for lentivirus production. shRNA-expressing lentiviral vectors were transduced into OT-II T cells activated with anti-CD3 and anti-CD28. Infected T cells were cultured in the presence of interleukin-2 (IL-2) and IL-7 for 7 to 10 days and sorted for Venus-positive OT-II T cells before the experiment. Immunoblotting was used to determine knockdown efficiencies. The following shRNA target sequences were used: kindlin-3, 5′-GAGAAGAAGGAGAAGAAGA (436 to 453); NDR1, 5′-ACAGAGTTTCTTCGATTAA (220 to 238); and RAPL, 5′-GGAATCTTCAGAGCAAACA (92 to 110). The production of lentiviral vectors containing the cDNA for wild-type or kinase-deficient (K59R) Mst1 was performed in a lentivirus vector driven by the EF promoter. Lentiviral vectors containing wild-type or kinase-deficient (K59R) Mst1 were transduced into Mst1/2 DKO OT-II T cells, and ectopically expressing T cells were sorted as described above. Lentivirus production and transduction of wild-type NDR1 or kinase-deficient (D212A) NDR1 into OT-II T cells were also performed as described above.

### Bone marrow transfer of the Rap affinity probe.

A GFP-tagged Rap affinity probe (GFP-RalGDS-RBD) expressed in a lentivirus vector with Venus was used for lentivirus production. The lentiviral vector was introduced into hematopoietic stem cells (CD34^−^ Kit^+^ Sca1^+^ lin^−^) isolated from the bone marrow of OT-II mice, which were then transferred into irradiated C57BL/6 mice (46). After 2 months, OT-II T cells expressing moderate levels of Venus were sorted from the spleen and lymph nodes of bone marrow-transferred mice and used for the experiments. Isolated OT-II T cells expressing the Rap affinity probe exhibited mature IS formation comparable to those without the probe.

### Immunostaining.

OT-II T cells were applied onto a supported planar lipid bilayer (SPLB) displaying CF568-labeled ICAM-1 and ATTO425-labeled StAv complexed with monobiotinylated I-A^b^ loaded with OVA peptide. Thirty minutes after incubation at 37°C, cells were fixed with 4% paraformaldehyde, permeabilized with 0.2% Triton X-100, and stained with the appropriate antibodies for 30 min, followed by secondary fluorophore-conjugated Abs for 30 min, and then they were washed with 0.1% BSA in PBS. Images were obtained using a confocal laser-scanning microscope (LSM700; Carl Zeiss) in z stack mode. Captured images were analyzed with ImageJ (http://rsb.info.nih.gov/ij/), Volocity (Improvision), and Prism (GraphPad Software).

### Imaging of T-DC interactions.

Two-photon imaging of T-DC interactions in sliced lymph nodes was performed as described previously (32, 67), with modifications. CD11c^+^ bone marrow-derived DCs (BMDCs) were stimulated overnight with lipopolysaccharide (LPS; 1 μg/ml). Labeled OVA-pulsed BMDCs (0.2 μM OVA_323–339_, 5 × 10^5^ cells) were subcutaneously injected into the front paw of C57BL/6 mice. The dose of OVA peptide was titrated and chosen to induce arrest adhesion of OT-II T cells as determined by intravital two-photon imaging. Twenty-four hours after injection, brachial LNs were isolated for the preparation of sliced lymph nodes. OT-II T cells isolated from wild-type and Mst1/2 DKO mice were labeled with CFSE and CMTMR, respectively, and combined at an equal ratio before being applied to the cut LNs. Thirty minutes after incubation at 37°C under 5% CO_2_ in RPMI 1640 supplemented with 1% FCS, the LN slices were examined using two-photon imaging (FV1000MPE; Olympus). Images were analyzed using Volocity software (Improvision). In some experiments, T cells were applied in the presence of anti-LFA-1 MAb (KBA) or a control antibody (67). DCs without antigen loading were used as a negative control for antigen-specific interactions.

## Supplementary Material

Supplemental material
