# Supplementary material for: NDR1-Dependent Regulation of Kindlin-3 Controls High-Affinity LFA-1 Binding and Immune Synapse Organization
Source: Mol Cell Biol. 2017 Mar 31;37(8):e00424-16. doi: 10.1128/MCB.00424-16 (PMC5376635; doi:10.1128/MCB.00424-16)
Supplement: Supplemental material [file supp_37_8_e00424-16__index.html]

NDR1-Dependent Regulation of Kindlin-3 Controls High-Affinity LFA-1 Binding and Immune Synapse Organization — Supplemental material 

# NDR1-Dependent Regulation of Kindlin-3 Controls High-Affinity LFA-1 Binding and Immune Synapse Organization

## Supplemental material

- Supplemental file 1 -

  Fig. S1 (Single-molecule measurement of ICAM-1), S2 (Effect of Mst1/2 and RAPL on proliferation, IS formation, and T-DC interactions), S3 (Validation of GFP-Rap affinity probe and kindlin-3 staining), S4 (Effect of RAPL knockdown on IS), S5 (Effect of Mn2+ on IS formation), and S6 (Supporting evidence of NDR1–kindlin-3 interaction) and legends to Videos S1 to S9

  PDF, 1.9M
- Supplemental file 2 -

  Video S1 (Single-molecule imaging of ICAM-1)

  MOV, 648K
- Supplemental file 3 -

  Video S2 (Single-molecule imaging of ICAM-1)

  MOV, 7.4M
- Supplemental file 4 -

  Video S3 (Single-molecule imaging of ICAM-1)

  MOV, 8.2M
- Supplemental file 5 -

  Video S4 (Time-lapse imaging of Rap1-GTP and LFA-1–bound single-molecular ICAM-1)

  MOV, 597K
- Supplemental file 6 -

  Video S5 (Time-lapse imaging of Rap1-GTP and pMHC microclusters)

  MOV, 376K
- Supplemental file 7 -

  Video S6 (Time-lapse imaging of GFP-kindlin3 and LFA-1–bound single-molecular ICAM-1)

  MOV, 164K
- Supplemental file 8 -

  Video S7 (Two-photon imaging of OT-II T cell interactions)

  MOV, 85K
- Supplemental file 9 -

  Video S8 (Two-photon imaging of OT-II T cell interactions)

  MOV, 62K
- Supplemental file 10 -

  Video S9 (Two-photon imaging of interactions between OT-II T cells and OVA-pulsed dendritic cells)

  MOV, 156K
