# Supplementary material for: NDR1-Dependent Regulation of Kindlin-3 Controls High-Affinity LFA-1 Binding and Immune Synapse Organization
Source: Mol Cell Biol. 2017 Mar 31;37(8):e00424-16. doi: 10.1128/MCB.00424-16 (PMC5376635; doi:10.1128/MCB.00424-16)
Supplement: Supplemental material [file MCB.00424-16_zmb999101463s1.pdf]

# Figure Supplement 1

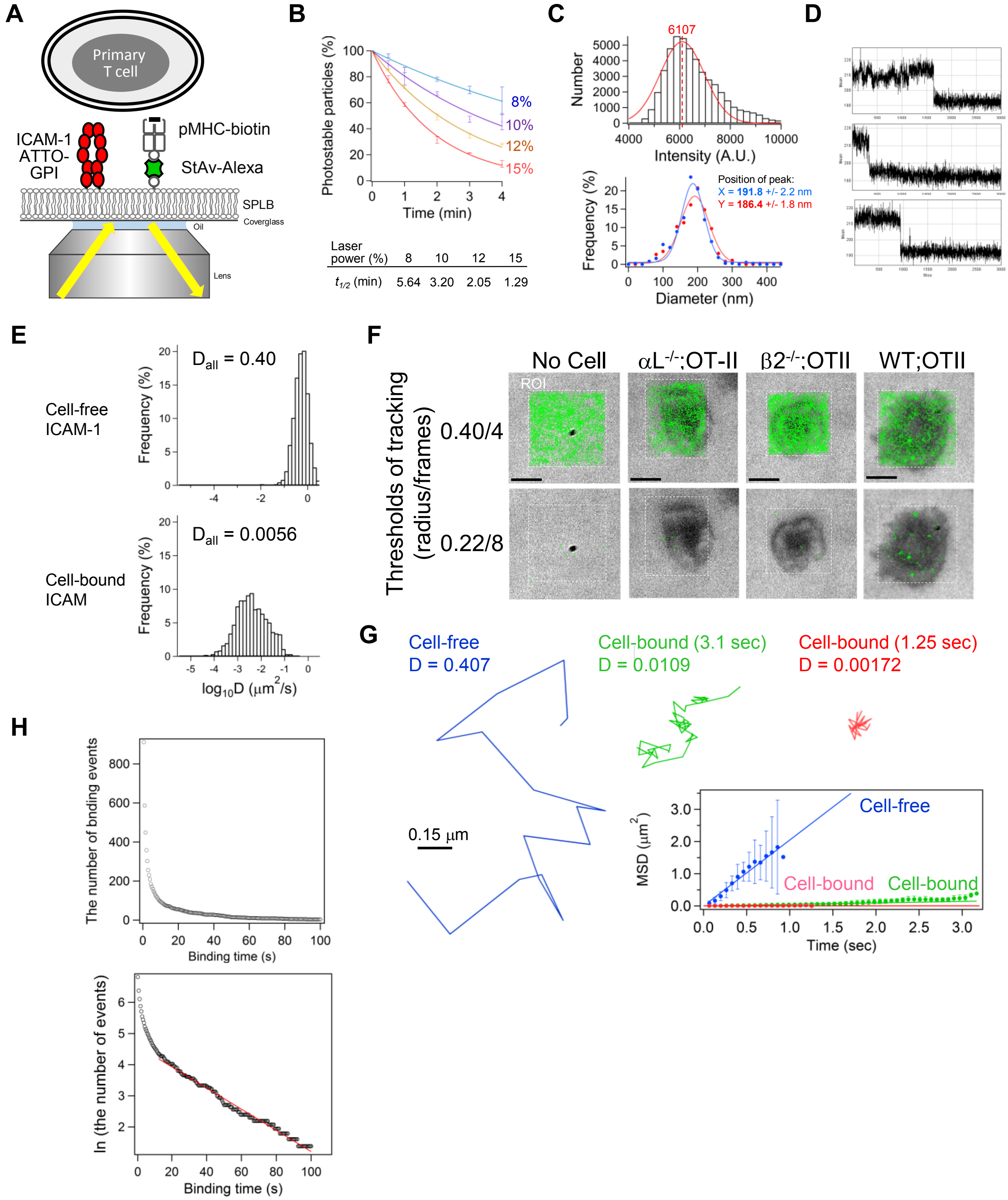

# Figure Supplement 2

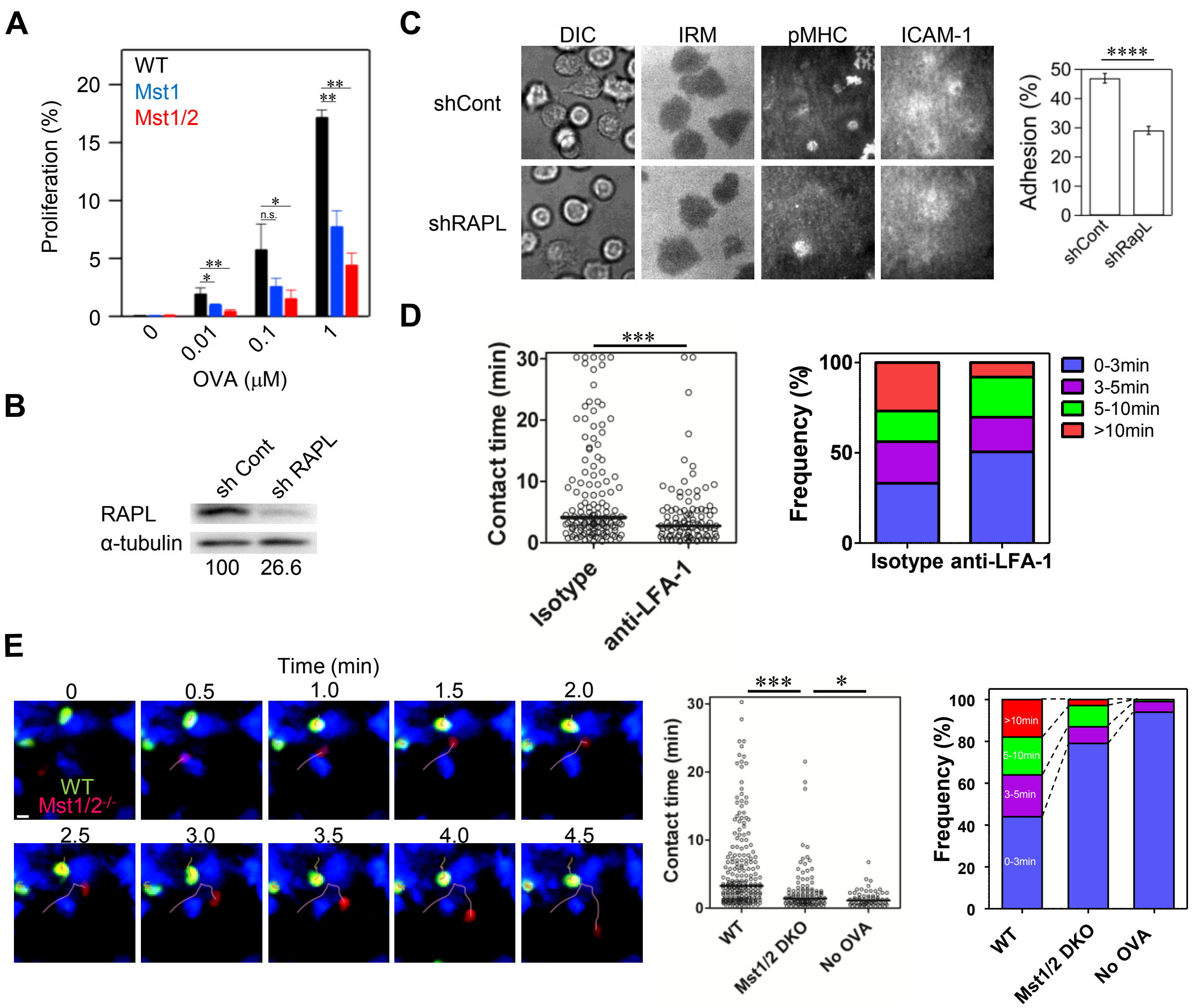

# Figure Supplement 3

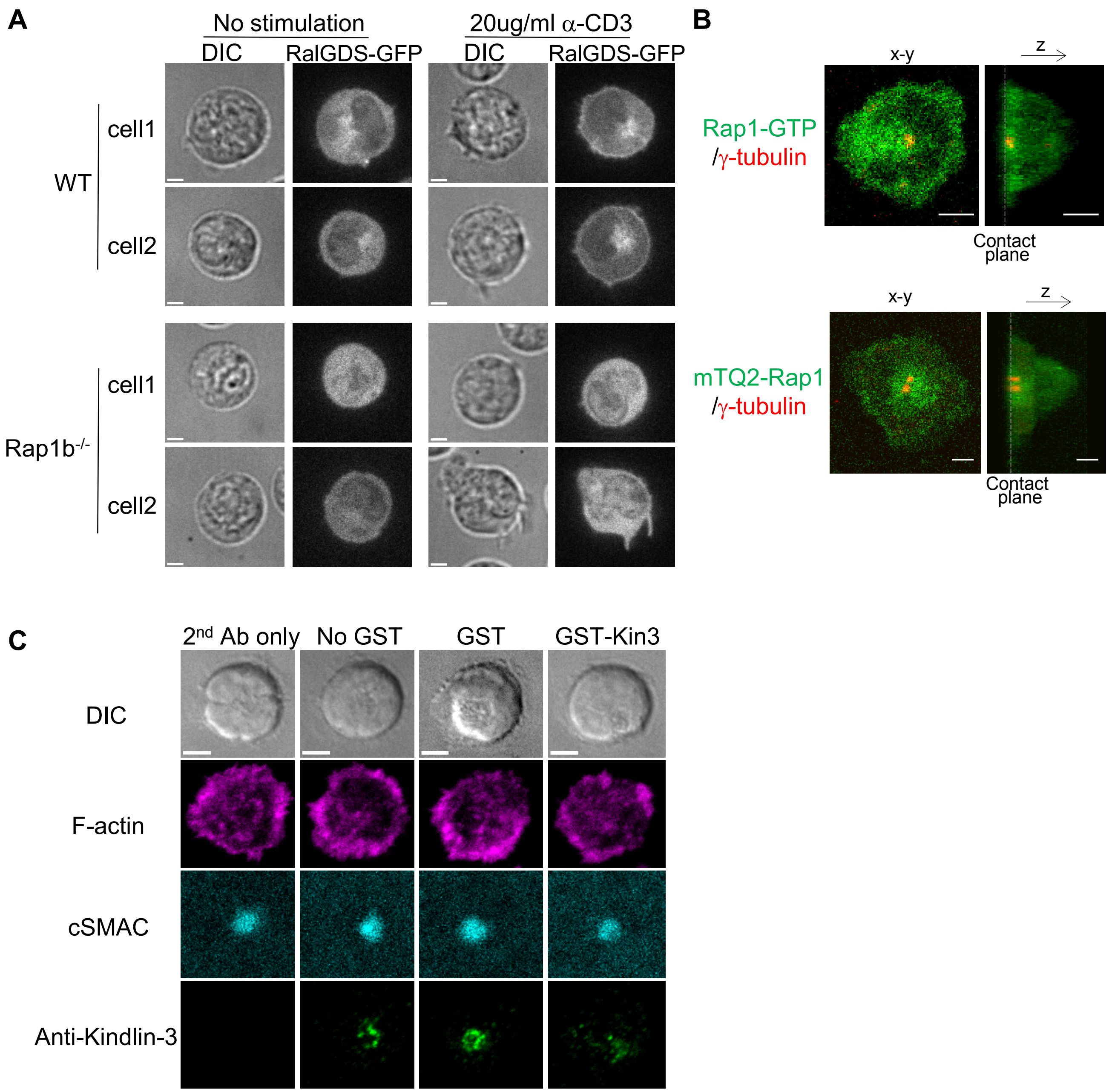

# Figure Supplement 4

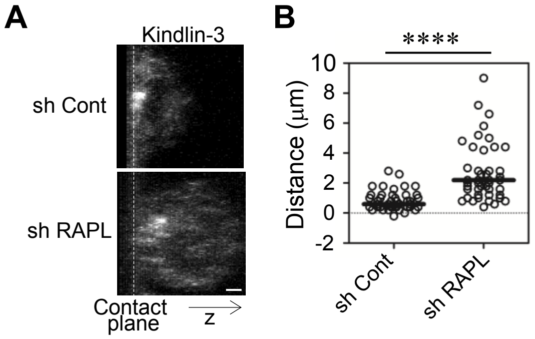

# Figure Supplement 5

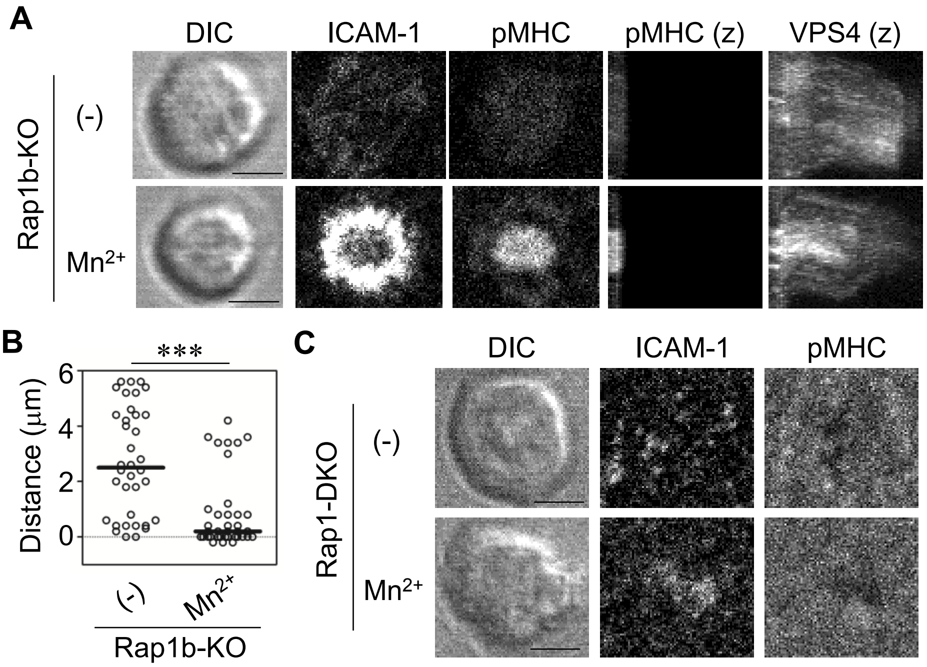

# Figure Supplement 6

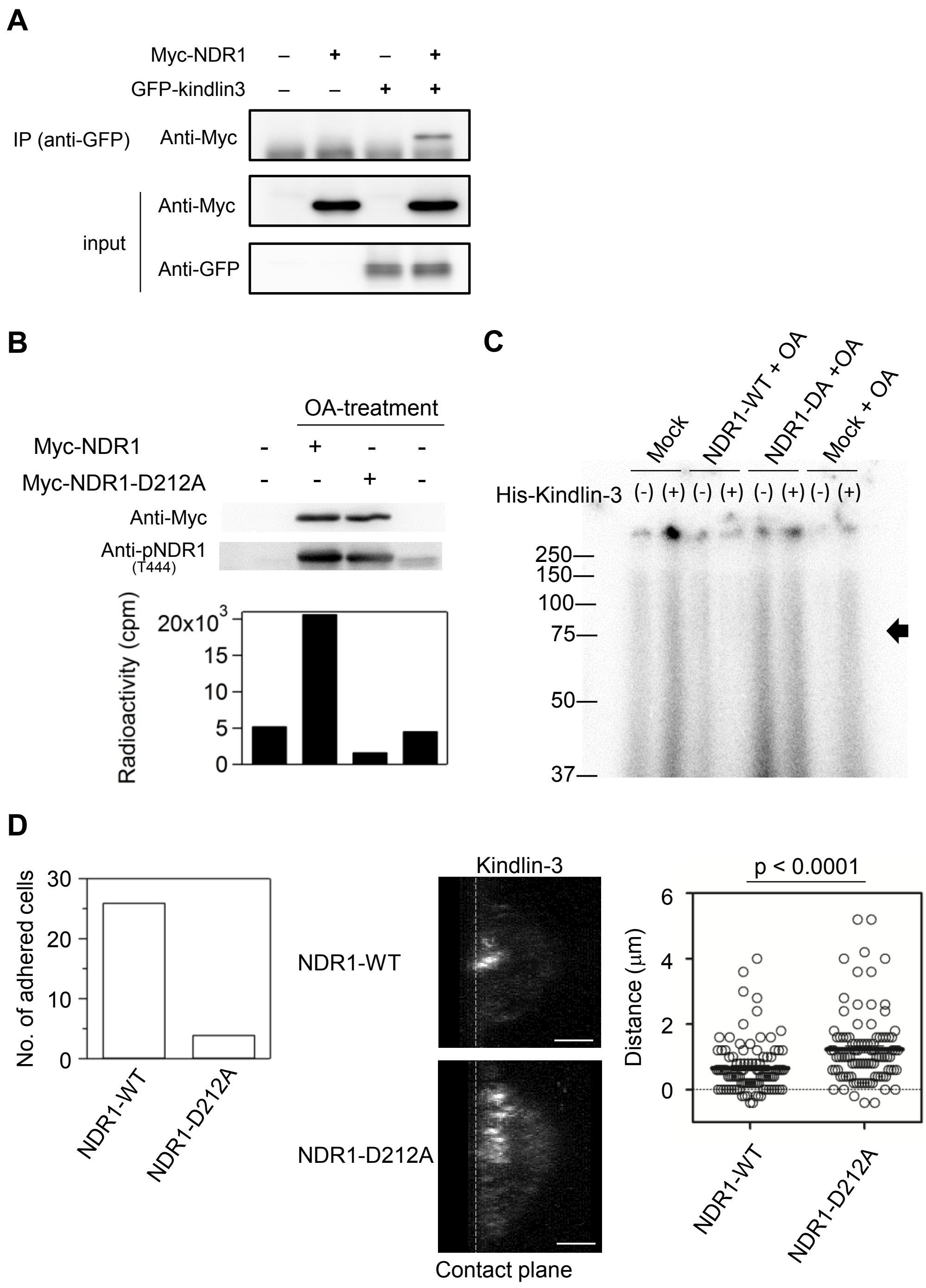

## SUPPLEMENTARY FIGURE LEGENDS

**Figure S1. Single-molecule measurement of ICAM-1.**

(**A**) Schematic representation of the experimental system. (**B**) Photostability of ICAM-1-ATTO647N. ICAM-1-ATTO647N-GPI displayed on supported planar lipid bilayers (SPLB) was dried on coverslips and excited at various outputs with a 641 nm solid-state laser (100 mW, Andor). Images were captured at indicated time points. Percentages of photo-resistant particles were calculated using: Photo-resistant particles (%) = 100 × (the number of residual bright spots) / (the number of bright spots before exposure). The bottom table indicates $t_{1/2}$ (min) of ICAM-1–ATTO647N excited at different laser outputs. (**C**) Distribution of intensities of ICAM-1–ATTO647N on SPLB. The red line indicates the best fitting curve using Gaussian function (top panel). Distribution of spot diameters. The values of the position of the center of the peak are near the diffraction limit of excitation wavelength (641 nm) (bottom panel). (**D**) Single-step photobleaching of ICAM-1–ATTO647N during laser exposure. (**E**) Histograms of the diffusion coefficients of single-molecule ICAM-1 (top, cell-free area) (bottom, cell-bound area of naïve OT-II $CD4^+$ T cells). $D_{\mathrm{all}}$ indicates diffusion coefficients calculated from all trajectories combined in cell-free and cell-bound areas. (**F**) Optimization of the thresholds of automated tracking of ICAM-1 in IS of wild-type and LFA-1–deficient OT-II T cells on SPLB presenting ICAM-1 and pMHC. Trajectories acquired with G-count software using tracking settings with search radii of 0.4 and 0.22 μm in 4 and 8 consecutive frames, respectively. Note that the latter setting extracted slow-moving ICAM-1 tracks, while very few tracks were seen in αL- or β2-deficient OT-II T cells. Scale bar: 2.5 μm. (**G**) Examples of trajectories of free, bound ICAM-1, and MSD vs. time interval curves. Individual trajectories were analyzed by generating mean-square displacement (MSD) curves to obtain the diffusion coefficient D. Trajectories with more than 10 time points

were retained and MSD was calculated as the average of the square of the molecular distance traveled during a given time interval, and the expectation value is over all pairs of time points separated by a time interval in each molecular track. MSD vs. time interval curves were generated for all possible time intervals. The slope of the linear fit that gave the maximum correlation was determined. The relationship MSD = $4Dt$ (t is the time interval) was used to derive the diffusion coefficient $D$. (**H**) Histogram of lifetimes of ICAM-1 binding in IS. (top) The lifetime histogram of ICAM-1 binding (n= 912). (bottom) The semilogarithmic plot of the lifetime histogram. When it was linear-fitted by a exponential function for long binding events, the dissociation rate constants ($k_{off}$) was 0.036 $sec^{-1}$ ($r^2 = 0.99$).

**Figure S2. The effect of Mst1/2 and RAPL on proliferation, IS formation, and T-DC interactions within lymph nodes**

(**A**) Antigen-dependent proliferation of T cells from wild-type (WT), *Mst1*$^{-/-}$ (Mst1), or *Mst1*$^{-/-}$;*Mst2*$^{-/-}$ (Mst1/2) mice. Fractions of proliferating cells were measured using flow cytometry 48 hours after co-cultured with bone marrow-derived dendritic cells supplemented with indicated concentrations of OVA antigen. *$p<0.05$, **$p<0.01$, n.s.: not significant. (**B**) Knockdown of RAPL in OT-II T cell blasts. Protein levels of RAPL in cultured OT-II T cells with control shRNA (sh Cont) and RAPL-specific shRNA (sh RAPL). Values at the bottom indicate the percentage of RAPL normalized to α-tubulin expression. (**C**) Representative images of DIC, IRM, pMHC, and ICAM-1 of control and RAPL knockdown T cell blasts IS. Attachment frequency of control and RAPL-knockdown T cells shown right. Percentages of attached cells were calculated based on IRM images from multiple fields. (**D**) Inhibition of antigen-induced T cell arrest on DC with anti–LFA-1 antibody. (left panel) Distribution of T–DC contact time during a 30 min imaging time frame with median contact time indicated by the bar. Median contact

duration for OVA-pulsed DC and T cells in the presence of anti-Rat IgG isotype control (Isotype, 4.13 min) and LFA-1 blocking mAb KBA (anti–LFA-1, 2.75 min). ***p<0.001. (right panel) Profiles of T–DC contacts are categorized and shown. (**E**) Antigen-induced T cell arrest on DC was impaired in naïve Mst1/2 DKO OT-II T cells. (left panels) Time-lapse images and trajectories of naïve wild-type T cells (WT, green), Mst1/2 DKO T cells (red), and OVA-pulsed dendritic cells (DC, blue) in lymphoid tissues. Scale bar: 5 µm. (middle panel) Distribution of T–DC contact time during a 30 min imaging time frame. Median contact time of OVA-pulsed DC with wild-type (WT, 3.25 min) and Mst1/2 DKO T cells (1.38 min), or wild-type T cells with non-pulsed DC (No OVA, 1.13 min). ***p<0.0001. *p<0.05. (right panel) Profiles of T-DC contacts categories are shown.

**Figure S3. Validation of GFP-Rap affinity probe and kindlin-3 staining**

(**A**) Confocal images of $CD4^+$ T cells harboring the Rap affinity probe (GFP-RalGDS-RBD), from wild-type or Rap1b-deficient (*CD4-Cre;Rap1b$^{f/f}$*) mice. T cells were incubated with or without an anti-CD3 antibody (2C11, 20 µg/ml) for 5 min before capturing confocal images. The Rap affinity probe was diffusely distributed throughout the cytoplasm without stimulation, but upon anti-CD3 stimulation it was detected in the plasma membrane. The localization of the Rap affinity probe to the plasma membrane was not seen in Rap1b-deficient T cells. Scale bar: 2.5 µm. (**B**) Three-dimensional localization of γ-tubulin and the Rap affinity probe (RalGDS-RBD-GFP) or mTurquoise2 (mTQ2)-Rap1. Scale bar: 2.5 µm. **(C)** Specificity of the kindlin-3 antibody. IS of naïve OT-II T cells was immunostained with an anti–kindlin-3 antibody (Millipore ab2979) with or without GST-Kindlin3 (GST-Kin3, amino acids 494–548 as antigen). GST was used as a negative control. Localization of endogenous kindlin-3 near the cSMAC was largely abrogated with GST-Kindlin-3 treatment. Scale bar: 2.5 µm.

**Figure S4. Effect of RAPL knockdown on IS.**

(**A**) 3D images (z) of kindlin-3 in cultured T cells treated with control shRNA (sh Cont) and RAPL-specific shRNA (sh RAPL). (**B**) Quantification of the distance of the peak intensity of kindlin-3 from the IS contact plane. ****p<0.0001. Scale bar: 2.5 μm.

**Figure S5. Effect of $Mn^{2+}$ on IS formation of Rap1b-deficient and and Rap1 DKO OT-II T cells**

(**A**) IS formation of Rap1b-deficient (Rap1b-KO) OT-II T cells on lipid bilayers presenting pMHC and ICAM-1 in medium containing non-stimulatory concentrations of cations, 0.5 mM $Ca^{2+}$, and 0.5 mM $MgCl_2$ (-), or stimulatory 1 mM $MnCl_2$ ($Mn^{2+}$). T cells were fixed and immunostained for VPS4. En face views of DIC, ICAM-1, and pMHC, as well as side views (z) of pMHC and VPS4 in IS are shown. Scale bar: 2.5 μm. Note increased intensities of pMHC and ICAM-1 in the presence of $Mn^{2+}$. (**B**) Quantification of the distance of peak intensities of VPS4 in IS of Rap1b-deficient OT-II T cells. ***p<0.0001. (**C**) The effect of $Mn^{2+}$ on IS formation in Rap1 DKO OT-II T cells. Cells were treated as above. Images of DIC, ICAM-1, and pMHC are shown. Scale bar: 2.5 μm.

**Figure S6. Supporting evidence of NDR1-Kindlin-3 interaction and the effect of kinase activity of NDR1 on Kindlin-3**

(**A**) Association of NDR1 and kindlin-3. 293T cells transfected with GFP-kindlin-3 and Myc-NDR1 as indicated were subjected to immunoprecipitation of GFP-kindlin-3 using anti-GFP antibody followed by immunoblotting for Myc-NDR1 using anti-Myc antibody.

(**B**) In vitro kinase assays of NDR1. HEK293T cells transfected with Myc tagged wild-type NDR1 and a kinase-deficient mutant (D212A) were treated with 1 µM okadaic acid for 1hr or left untreated, and lysed with NP-40 lysis buffer and subjected for immunoprecipitation with anti-Myc antibody and protein G Sepharose. In vitro kinas assay was performed as described using control substrate peptide (Stegert, M. R. et. al. *Mol. Cell. Biol.* (2005) 25:1109-11029) and measured incorporated $^{32}P$ using TRI-CARB 2700TR (Packard) liquid scintillation analyzer.

(**C**) In vitro kinase assay was performed as above using His-kindlin-3 as substrate and phosphorylation of kindlin-3 was analyzed by SDS-PAGE and autoradiography (FLA-2000, Fujifilm). Arrow indicate expected molecular weight of His-kindlin-3

(**D**) Effect of overexpression of a kinase-deficient NDR1 mutant (D212A) on kindlin-3 localization. OT-II T cells expressing wild-type NDR1 (NDR1-WT) and kinase-deficient mutant D212A ($3.5 \times 10^3$ cells each) were incubated on lipid bilayers presenting pMHC and ICAM-1 for 30 min and fixed for immunostaining with anti-kindlin-3 antibody. (left) The numbers of adherent cells from twenty imaging fields. (middle) Three-dimensional localization of kindlin-3 in NDR1-WT or NDR1-D212A–expressing OT-II T cells. Scale bar: 2.5 µm. (right) Distances of peak intensities from the contact plane of kindlin-3 are shown.

**Video 1**

Single-molecule imaging of ICAM-1 in the absence of cells. Time-lapse images taken at a 33 msec interval (6000 frames in total) were shortened to 3000 images (66 msec

interval). The first thousand frames are replayed at 30 frames/sec. Image size: 9.3 μm × 9.3 μm.

**Video 2.**

Single-molecule imaging of ICAM-1 in IS of naïve OT-II T cells. Time-lapse images taken at a 33 msec interval (6000 frames in total) were shortened to 3000 images (66 msec interval) and displayed at 30 frames/sec. Duplicate images with trajectories are shown on the right. Image size: 9.3 μm × 9.3 μm.

**Video 3.**

Single-molecule imaging of ICAM-1 in IS of OT-II T cell blasts, as shown in Supplemental Movie 1. Duplicate images with trajectories are shown on the right. Image size 10.9 μm × 10.9 μm.

**Video 4.**

Time-lapse imaging of Rap1-GTP (green) and LFA-1–bound single-molecular ICAM-1 (red) in IS of naïve OT-II T cells with a time interval of 5 sec. Image size: 10 μm × 10 μm.

**Video 5.**

Time-lapse imaging of Rap1-GTP (green) and pMHC microclusters (blue) in IS of naïve OT-II T cells taken at a 5 sec interval. Image size: 10 μm × 10 μm.

**Video 6**

Time-lapse imaging of GFP-Kindlin3 and LFA-1–bound single-molecular ICAM-1 in IS of OT-II T cell blasts taken at a 10 sec interval. Image size 17.5 μm × 17.5 μm.

**Video 7**

Two-photon imaging of OT-II T cell interactions in the presence of isotype control Rat IgG (red) and OVA-pulsed dendritic cells (blue) in sliced lymph nodes with a time interval of 15 sec. Image size 36.7 μm × 36.7 μm.

**Video 8**

Two-photon imaging of OT-II T cell interactions in the presence of a blocking anti–LFA-1 monoclonal Ab (KBA, red) and OVA-pulsed dendritic cells (blue) in sliced lymph nodes with a time interval of 15 sec. Image size 25 μm × 25 μm.

**Video 9**

Two-photon imaging of the interactions between OT-II T cells (WT: green, Mst1/2 DKO red) and OVA-pulsed dendritic cells (blue) in sliced lymph nodes with a time interval of 15 sec. Image size 50 μm × 50 μm.
